# Enhanced oxidative phosphorylation, re-organized intracellular signaling, and epigenetic de-silencing as revealed by oligodendrocyte translatome analysis after contusive spinal cord injury

**DOI:** 10.1038/s41598-023-48425-6

**Published:** 2023-12-01

**Authors:** Michael D. Forston, George Z. Wei, Julia H. Chariker, Tyler Stephenson, Kariena Andres, Charles Glover, Eric C. Rouchka, Scott R. Whittemore, Michal Hetman

**Affiliations:** 1 Kentucky Spinal Cord Injury Research Center, University of Louisville School of Medicine, Louisville, KY 40202 USA; 2 Department of Neurological Surgery, University of Louisville School of Medicine, Louisville, KY 40202 USA; 3 Department of Anatomical Sciences & Neurobiology, University of Louisville School of Medicine, Louisville, KY 40202 USA; 4 Department of Pharmacology & Toxicology, University of Louisville School of Medicine, Louisville, KY 40202 USA; 5 MD/PhD Program, University of Louisville School of Medicine, Louisville, KY 40202 USA; 6 Kentucky IDeA Networks of Biomedical Research Excellence (KY INBRE) Bioinformatics Core, University of Louisville, Louisville, KY 40202 USA; 7 Neuroscience Training, University Louisville School of Medicine, Louisville, KY 40202 USA; 8 Department of Biochemistry and Molecular Genetics, University of Louisville School of Medicine, Louisville, KY 40202 USA

**Keywords:** Spinal cord diseases, White matter disease, Cell death in the nervous system, Myelin biology and repair, Oligodendrocyte

## Abstract

Reducing the loss of oligodendrocytes (OLs) is a major goal for neuroprotection after spinal cord injury (SCI). Therefore, the OL translatome was determined in Ribotag:Plp1-CreERT2 mice at 2, 10, and 42 days after moderate contusive T9 SCI. At 2 and 42 days, mitochondrial respiration- or actin cytoskeleton/cell junction/cell adhesion mRNAs were upregulated or downregulated, respectively. The latter effect suggests myelin sheath loss/morphological simplification which is consistent with downregulation of cholesterol biosynthesis transcripts on days 10 and 42. Various regulators of pro-survival-, cell death-, and/or oxidative stress response pathways showed peak expression acutely, on day 2. Many acutely upregulated OL genes are part of the repressive SUZ12/PRC2 operon suggesting that epigenetic de-silencing contributes to SCI effects on OL gene expression. Acute OL upregulation of the iron oxidoreductase *Steap3* was confirmed at the protein level and replicated in cultured OLs treated with the mitochondrial uncoupler FCCP. Hence, STEAP3 upregulation may mark mitochondrial dysfunction. Taken together, in SCI-challenged OLs, acute and subchronic enhancement of mitochondrial respiration may be driven by axonal loss and subsequent myelin sheath degeneration. Acutely, the OL switch to oxidative phosphorylation may lead to oxidative stress that is further amplified by upregulation of such enzymes as STEAP3.

## Introduction

Contusive spinal cord injury (SCI) has a complex pathogenesis that involves time-dependent components including primary and secondary injuries as well as post-injury remodeling and plasticity^1^. SCI associated white matter damage (WMD) is a major driver of functional deficits below the level of injury^2,3^. Death of OLs contributes to WMD post-SCI^4,5^. However, few OL-expressed genes such as *p75/Ngfr*^6^, *Bax*^7^ or *Klk8*^8^ have been implicated as mediators of SCI-induced OL death/WMD. This is at least partly due to limited insight into the OL gene expression programs after SCI.

Single cell (sc) RNASeq technology has been recently applied to study the SCI transcriptomic response at the cellular level^9,10^. Specifically, scRNASeq-enabled transcriptomic phenotyping revealed region-specific changes in OL subtype content at chronic timepoints after hemisection or contusion SCI^9^. However, few SCI-associated OL gene expression changes were detected^9^. Importantly, the currently available scRNAseq technology has significant limitations. Its reliance on successful sorting of suspensions of viable cells is a problem when dealing with cells that have complex morphologies, spatially-regulated transcriptomes and/or sustained significant damage due to injury^11^. Additional challenges include limited depths/low sensitivity, high level of stochastic variability of single cell transcriptomes, ambiguity in interpreting negative signals, and data set contamination with highly expressed mRNAs that were released from other cells that lysed during sample preparation and/or sorting^11,12^. Last, but not least, scRNASeq is focused on overall cellular mRNA levels. Therefore, scRNASeq data lack information on gene expression regulation at the level of protein synthesis. Yet, such post-transcriptional regulation, that is present in neurons and OLs, plays a major role in response of those cells to pathologies^13–15^.

Isolation of translating ribosomes and quantification of their associated mRNAs offers insight into the cell translatome and accounts for translation initiation, which is the critical regulatory step of protein synthesis^13–15^. The Ribotag technology enables analysis of cell type-specific translatomes in whole animal studies^16^. In Ribotag mice, cell type-specific expression of the Cre recombinase results in removal of a stop codon to produce the RPL22 protein with a hemagglutinin (HA) tag at the C-terminus^16^. The large ribosomal subunit that contains RPL22-HA associates with the small ribosomal subunit during successful translation initiation, which enables immunoaffinity isolation of translating ribosomes by targeting the HA tag. Then, cell type-specific translatomes are determined by RNASeq^16^. Ribotag has been successfully applied to study astrocyte- or macrophage translatomes after contusive thoracic SCI^17,18^.

The current study has been initiated to determine the translatome of mature OLs at various stages of recovery following moderate contusive SCI at the thoracic segment (T)9 level. Ribotag identified hundreds of differentially expressed genes in SCI-challenged OLs. These newly described gene expression landscapes implicate axonal disconnection and loss of myelin sheaths as major drivers of the acute OL gene expression response to SCI. Unexpectedly, similar factors may contribute to OL gene expression regulation in the subchronic phase of the recovery.

## Results

### Isolation and sequencing of the OL translatome from the spinal cord

**Figure 1:**
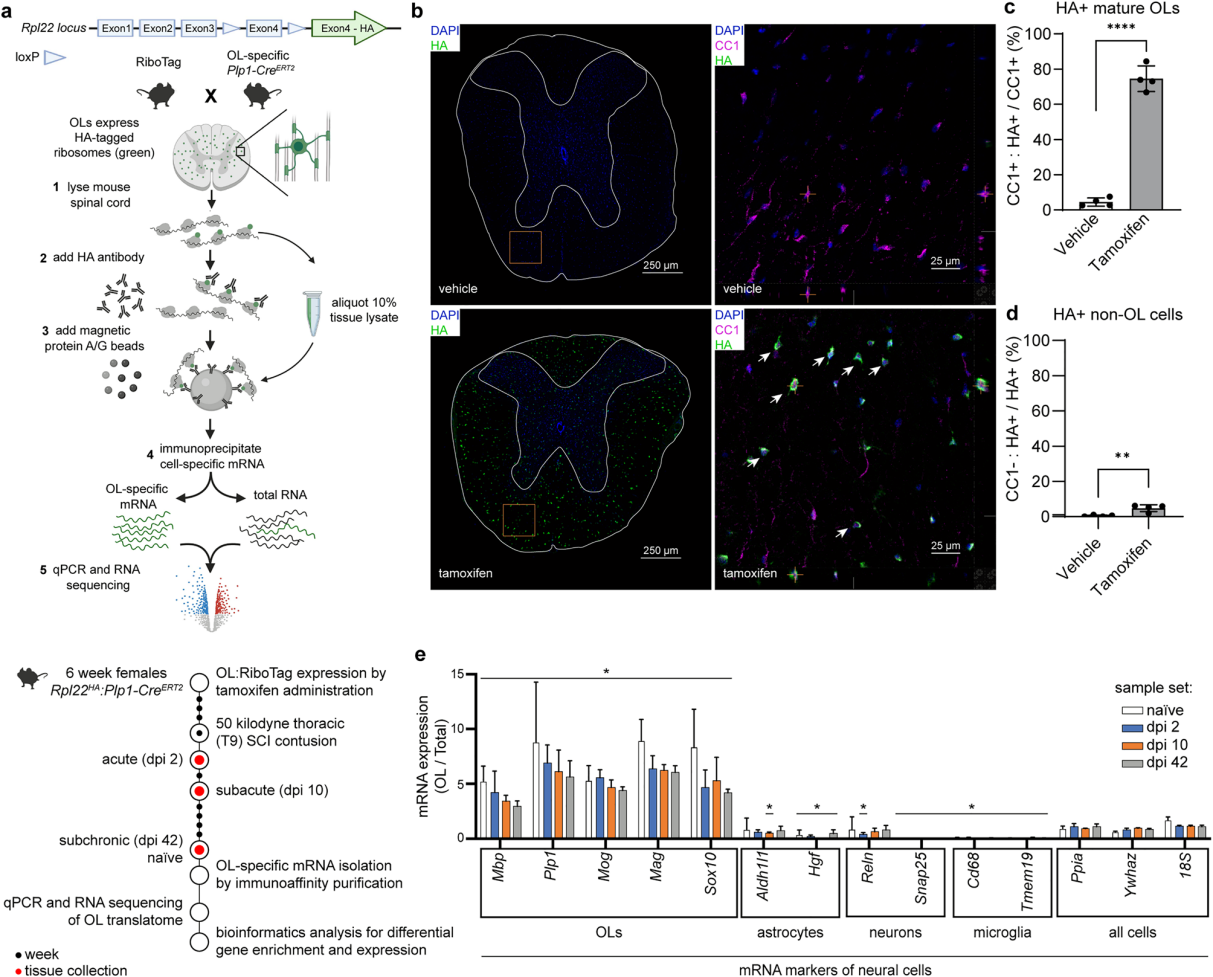
Successful induction of OL-Ribotag and isolation of the OL translatome. (**a**) Design of the current OL-Ribotag study. OL-Ribotag mice are generated by combining the conditional Rpl22-HA knock in (Ribotag) with Plp1-driven CreERT2. After tamoxifen treatment, RPL22-HA is expressed selectively in mature OLs enabling immunoaffinity purification of their translatomes. OL or all cell (total RNA) mRNA expression is measured using qPCR or RNASeq. After normalization, OL enriched expression can be calculated as a fold total RNA sample. (**b**–**d**) Co-immunostaining for HA and the OL-specific CC1 epitope in thoracic spinal cord of vehicle- or tamoxifen-induced OL-Ribotag mice. Section outline and grey matter border is marked on low power magnification images; frames indicate areas that were imaged under high power magnification. Note the appearance of many HA^+^ cells in the white matter of tamoxifen-treated animals. Most of those cells were also CC1^+^ (arrowheads). Cell counting in the white matter showed efficient ((**c**) %CC1^+^ cells that are HA^+^) and specific ((**d**) %HA^+^ cells that are CC1^–^) induction of RPL22-HA in tamoxifen-treated OL-Ribotag mice. (**e**) Tamoxifen-induced OL-Ribotag mice received SCI and their contused spinal cord tissue was used for OL translatome isolation at the indicated days post-injury (dpi). Cell type marker transcripts were determined in OL and corresponding total RNA samples by qPCR. All cell-expressed housekeeping genes were used for normalization. Note the OL enrichment of OL marker mRNAs across all experimental groups. Conversely, several non-OL cell marker mRNAs were OL-depleted. Data on graphs represent averages ± SD from 3 individual animals (**c**,**d**) or 3 biological samples pooled from 2 female mice each (**e**); *p < 0.05; **p < 0.01; ***p < 0.001 Mann–Whitney *u*-test).

OL-Ribotag mice were treated with tamoxifen to activate Cre-mediated recombination of the *Rpl22*^*fl(STOP)fl-HA*^ allele (Fig. 1a). Four weeks later, HA immunostaining was observed in 75% or 5% of CC1^+^ or CC1^-^ cells throughout the thoracic spinal cord, respectively (Fig. 1b–d). Double-positive cells were present in the white and grey matter, as expected for mature, CC1^+^ OLs (Fig. 1b,c). Conversely, in vehicle-treated controls, HA^+^ cells were rare (< 2% or < 0.5% CC1 + or CC1- cells, respectively, Fig. 1b–d). Therefore, tamoxifen treatment resulted in efficient and OL-specific expression of RPL22-HA in OL-Ribotag mice.

Next, moderate contusive SCI (50 kilodyne (kdyn), T9) was performed in tamoxifen-induced OL-Ribotag mice and 5 mm spinal cord segments spanning the lesion site were collected at day post-injury (dpi) 2, 10, and 42. These time points were selected based on dynamics of spinal cord pathology after SCI including acute OL loss at the injury epicenter (dpi 2), peak of delayed OL apoptosis in the spared white matter (dpi 10) and limited remyelination (dpi 42)^4,5,19^. With the reported rostral-caudal lesion span of 1–1.5 mm after T9 50 kdyn IH SCI in young adult female mice^20^, each sample included the primary damage site, spared white matter at the injury epicenter, the injury penumbra, and uninjured regions both caudal and rostral to the epicenter. Uninjured, naïve OL-Ribotag mice were used as controls. Control and dpi 42 samples were collected on the same day, i.e*.* 10 weeks after starting tamoxifen induction (16 weeks of age). As most mature OLs survive for at least 8 months of mouse life^21^, stable levels of Ribotag labeling are expected over a 10-week post-induction period.

After isolation of total spinal cord polysomes, OL polysomes were immunoprecipitated using anti-HA antibody. To verify their successful isolation, qPCR for neural cell marker transcripts was performed. As expected for translatomes from cells that are estimated to make about 20% of all spinal cord cells^22,23^, the average enrichment of an OL marker mRNA reached 5.89 fold change (FC) total RNA control (Fig. 1e). Conversely, several astrocytic, neuronal or microglial marker transcripts were depleted from OL translatome samples (median FC 0.52, 0.36 or 0.1, respectively, Fig. 1e). Such a differential enrichment pattern was observed across all samples indicating successful isolation of the OL translatome from spinal cord tissue of OL-Ribotag mice.

**Figure 2:**
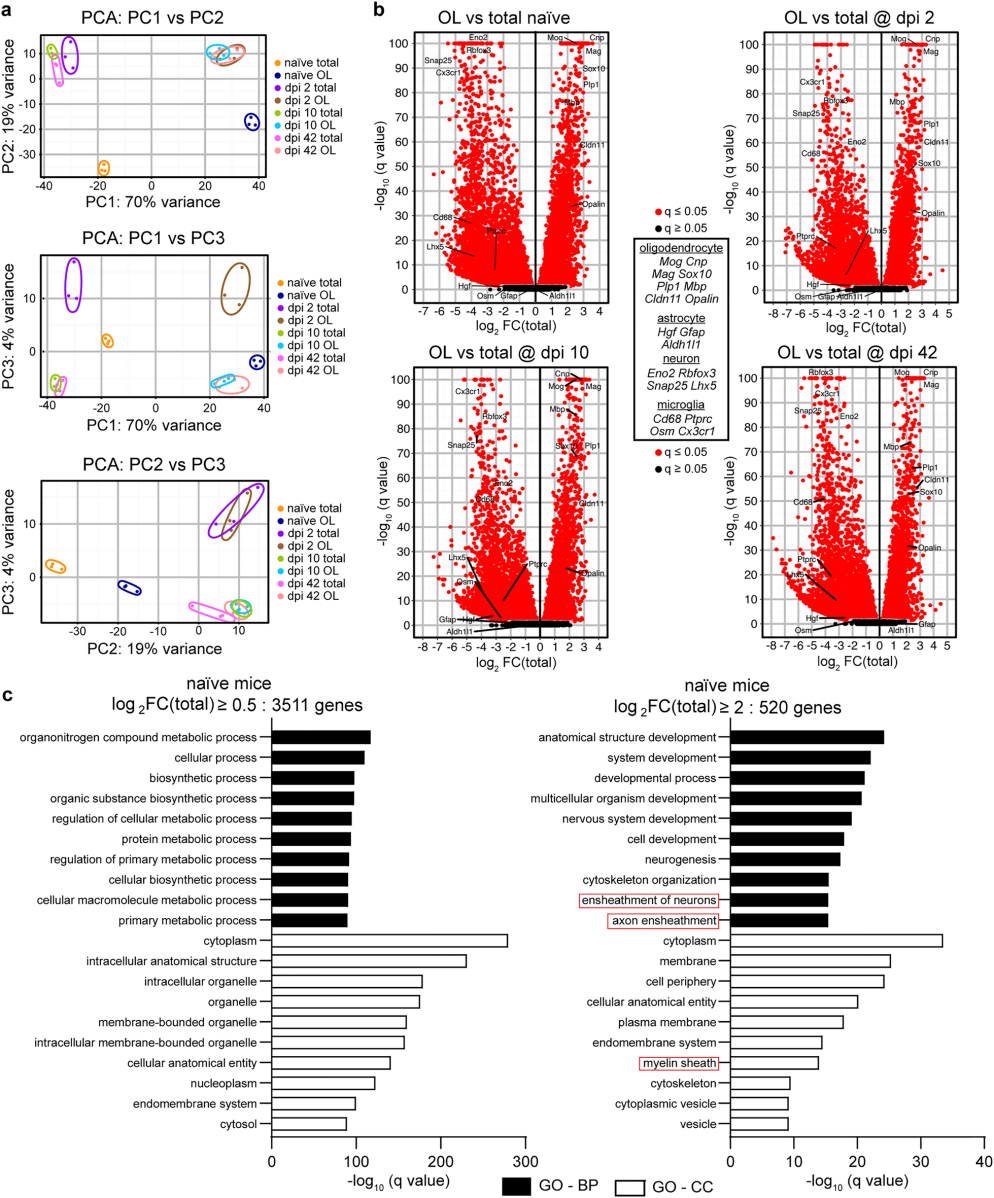
RNASeq confirms successful isolation of the OL translatome from intact or injured spinal cord tissue. Tamoxifen-induced OL-Ribotag mice received SCI and OL translatomes were immunopurified as described in Fig. 1. Following qPCR analysis of cell marker transcripts (Fig. 1e), mRNA expression in the OL translatome and corresponding total RNA samples was analyzed by RNASeq. (**a**) Principal component analysis (PCA) was performed across all samples and included mRNAs with a read count ≥ 10. Note that 70% of variance (PC1) was related to OL vs. total RNA origin followed by 19% (PC2) or 4% (PC3) due to effects of SCI across all time points or on dpi 2, respectively. (**b**) Volcano plots presenting all differentially expressed genes/mRNAs (DEGs) between OL vs. total RNA samples from the indicated groups. Note the significant OL enrichment or depletion of OL or microglia/neuron marker mRNAs across all groups, respectively. All OL DEGs are listed in Supplementary Table S1. (**c**) The top 10 most overrepresented gene ontology biological process (GO-BP) or cellular component (GO-CC) terms among OL-enriched mRNAs from naïve mice as determined by q value ranking. GOs related to OL function (red boxes) show a high level of overrepresentation when highly, but not moderately, enriched OL mRNAs are analyzed. Similar GO overrepresentation patterns emerged in OL DEGs from SCI samples (Supplementary Table S2).

RNASeq was then performed on all samples. The principal component analysis (PCA) of the resulting mRNA expression data revealed robust separation of samples that represented OL-enriched translatome vs. total RNA input with the PC1 accounting for 70% variance (Fig. 2a). Further separation was also evident including that between naïve and SCI samples (PC2, 19% variance, Fig. 2a) or dpi 2 vs. other samples (PC3, 4% variance, Fig. 2a).

Next, OL-enriched mRNAs were identified for each set of samples. As compared to total RNA inputs, OL enrichment of Log2FC(Total) > 0.5 was observed for 3,511, 3,302, 3,262, or 3,313 mRNAs in naïve, dpi 2, dpi 10 or dpi 42 samples, respectively (q < 0.05, Supplementary Table S1). While established mRNA markers of mature OLs were enriched, mRNA markers of neurons, astrocytes or microglia were depleted (Fig. 2b). Gene ontology term enrichment analysis (GO) was performed for OL-enriched mRNAs (Fig. 2c and Supplementary Table S2). The top enriched GO terms did not include OL-specific biological processes (BPs) or cellular components (CCs, Fig. 2c). However, OL-specific GO-BPs/CCs such as axon ensheathment, myelination, or myelin sheath were highly overrepresented when the analysis focused on 520 mRNAs with highly selective OL expression as defined by Log_2_FC(Total) > 2, q < 0.05 (Fig. 2c). A similar pattern of GO term enrichment was observed when analyzing OL-specific mRNAs from other samples sets including dpi 2, 10, or 42 (Supplementary Fig. S1 and Table S2). Therefore, OL translatomes were successfully isolated from both intact and injured spinal cords.

### Identification of differentially expressed OL genes after SCI

**Figure 3:**
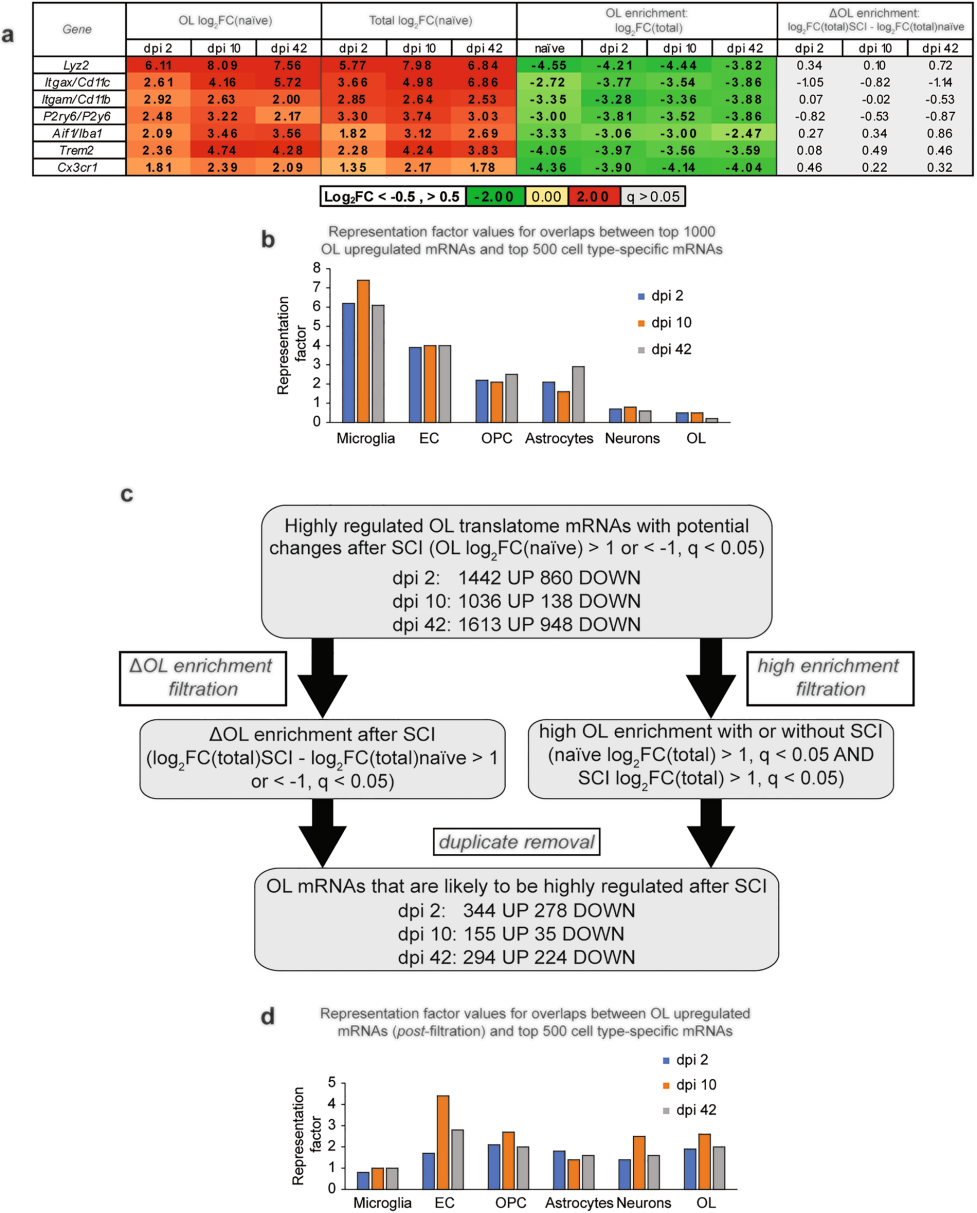
Determining OL gene expression response to SCI. Differential gene expression was analyzed in OL translatomes from SCI vs. naïve samples. (**a**,**b**) SCI OL translatome contamination with marker mRNAs of microglia/monocyte-derived macrophages. At each timepoint after SCI, the top 1000 highly upregulated transcripts (Log_2_FC/naïve/ > 1, q < 0.05) included several established markers of microglia and/or monocyte-derived macrophages (**a**). While sharply upregulated in total RNA samples, those microglial mRNAs were OL depleted and their OL de-enrichment was unaffected after SCI. (**b**) Significant overrepresentation of the top 500 microglia marker transcripts (Brain RnaSeq database) among OL-upregulated mRNAs after SCI, representation factor = 1 if the number of overlapping genes is as expected by a random chance (*OPC* oligodendrocyte precursor cells, *EC* endothelial cells). (**c**) Flow chart of the filtration procedure for high confidence identification of the SCI-upregulated component of the OL translatome. The two-arm filtration process was based on (i) differential OL enrichment after SCI or (ii) constantly high OL enrichment before and after SCI. In each case, OL expression analysis took into account changes in total RNA (see Text for more details, all identified highly regulated OL SCI DEGs are listed in the Supplementary Table S3). (**d**) After filtration, no overrepresentation of microglial marker mRNAs is present among OL mRNAs that are upregulated after SCI.

To compare SCI-mediated changes in OL-enriched translatomes, differentially expressed mRNAs were identified between OL SCI vs. OL naïve samples (|Log_2_FC/naïve/|> 1, q < 0.05). However, several well-established markers of microglia/monocyte-derived macrophages were identified as OL-upregulated after SCI (Fig. 3a). Those included such mRNAs as *Itgam/Cd11b*, *Cx3cr1*, and *Aif1/Iba1*, whose post-SCI protein expression has been confirmed in many studies to be microglia/macrophage-specific (as reviewed in Ref.^19,24^). In support of being expressed mainly by inflammatory cells, all those marker transcripts remained OL-depleted after SCI and their OL de-enrichment did not change significantly (Fig. 3a). Moreover, extensive overlaps were observed between SCI-upregulated mRNAs from OL translatomes and the top 500-microglia-enriched mRNAs (Fig. 3b). Such findings suggest that some mRNAs that are present in OL translatome represent a contamination from non-OL cells, as recently shown in heterologous culture systems^25^. Such a non-specific co-purification with cell type-tagged ribosomes may be particularly relevant acutely after CNS injury when tissue cellularity changes and some mRNAs such as those expressed by the inflammatory cells become extremely abundant^10^. To reduce interference by those potential contaminating transcripts, a two-arm filtration procedure was applied to differentially expressed mRNAs from OL translatomes (Fig. 3c, see “Material and methods” for further details). By taking into account not just the OL expression change but also differential- (arm 1) or constantly high (arm 2) OL enrichment, the filtration identified hundreds of mRNAs that represent high confidence components of OL gene expression response to SCI (Fig. 3c,d, Supplementary Table S3).

### Bioenergetic re-organization and reduced morphological complexity/connectivity as major components of OL-specific gene expression response to SCI

**Figure 4:**
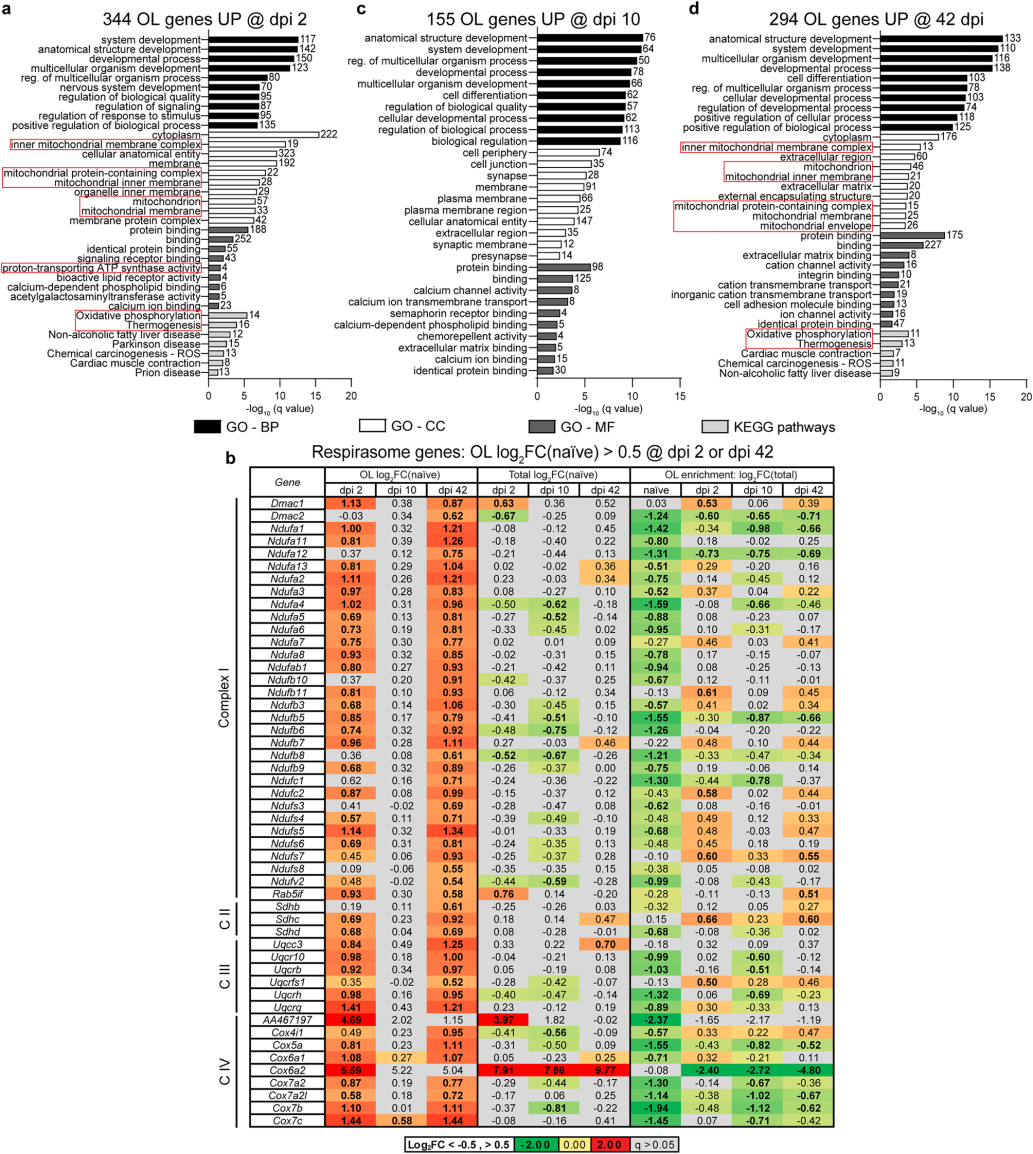
The OL response to SCI includes biphasic upregulation of mitochondrial respirasome genes. (**a**,**c**,**d**) Overrepresented GOs (top 10/category) among high confidence OL translatome mRNAs that are highly upregulated after SCI (Log_2_FC/naïve/ > 1, q < 0.05, and two-arm filtration /Fig. 3c/). Several GOs related to mitochondria are overrepresented on dpi 2 and 42, but not dpi 10 (red boxes). MF- molecular function. (**b**) Fifty out of 68 mitochondrial respirasome genes that are OL-expressed are also OL upregulated on dpi 2 and/or 42 (Log_2_FC/naïve/ > 0.5, q < 0.05, bold). In most cases, their total spinal cord expression is unaffected (q > 0.05, grey cells). Therefore, the biphasic upregulation of mitochondrial respirasome genes is OL-specific. In naïve mice, most of those upregulated genes are OL depleted (Log_2_FC/Total/ < − 0.5, q < 0.05, bold), which is consistent with reduced activity of oxidative phosphorylation in mature OLs.

Among 344 highly upregulated transcripts on dpi 2, the top-enriched GO-BP terms included several broad categories such as those related to development, nervous system development, regulation of biological quality or signaling (Fig. 4a and Supplementary Table S4). More specificity emerged among top enriched GO-CC, GO-MF, and KEGG pathway terms. Those included such mitochondrial function-associated GOs as inner mitochondrial membrane, mitochondrion, proton-transporting ATP synthase activity, oxidative phosphorylation, or thermogenesis. Interestingly, eight components of the mitochondrial respirasome were highly upregulated (Log_2_FC(naïve) > 1, Fig. 4b). Moreover, 30 additional respirasome genes were found when the OL upregulation threshold was lowered to Log_2_FC(naïve) > 0.5 (q < 0.05, Fig. 4b). Together, over 55% of 68 OL-expressed mitochondrial respirasome genes (GO:0005746) were upregulated with median Log_2_FC(naïve) = 0.87 (q < 0.05) suggesting a coordinated response to increase oxidative phosphorylation.

On dpi 10, 155 highly upregulated mRNAs showed greatest overrepresentation of several broad GO-BP terms that were related to development (Fig. 4c and Supplementary Table S4). In addition, enrichment of synapse-related GO-CCs was observed (Fig. 4c). Upregulation of synapse-associated transcripts could represent an attempt to re-establish OL-axonal synapses that were likely lost during the acute phase of SCI-associated axonal injury^26^. Development remained a top-enriched GO-BP theme among 294 highly upregulated mRNAs on dpi 42 (Fig. 4d and Supplementary Table S4). In addition, high enrichment of mitochondria-associated GOs was found including the GO-CC or KEGG pathway terms mitochondrial inner membrane, mitochondrion, oxidative phosphorylation and thermogenesis (Fig. 4d). On dpi 42, both the spectrum and scale of mitochondria-related mRNA upregulations appeared to be even greater than that on dpi 2. Specifically, 48 mitochondrial respirasome genes were upregulated in OLs on dpi 42 (Log_2_FC(naïve) > 0.5, q < 0.05, Fig. 4b) with median Log_2_FC(naïve) = 0.95). These data suggest that on dpi 42, OL metabolism is again reorganized to favor oxidative phosphorylation.

**Figure 5:**
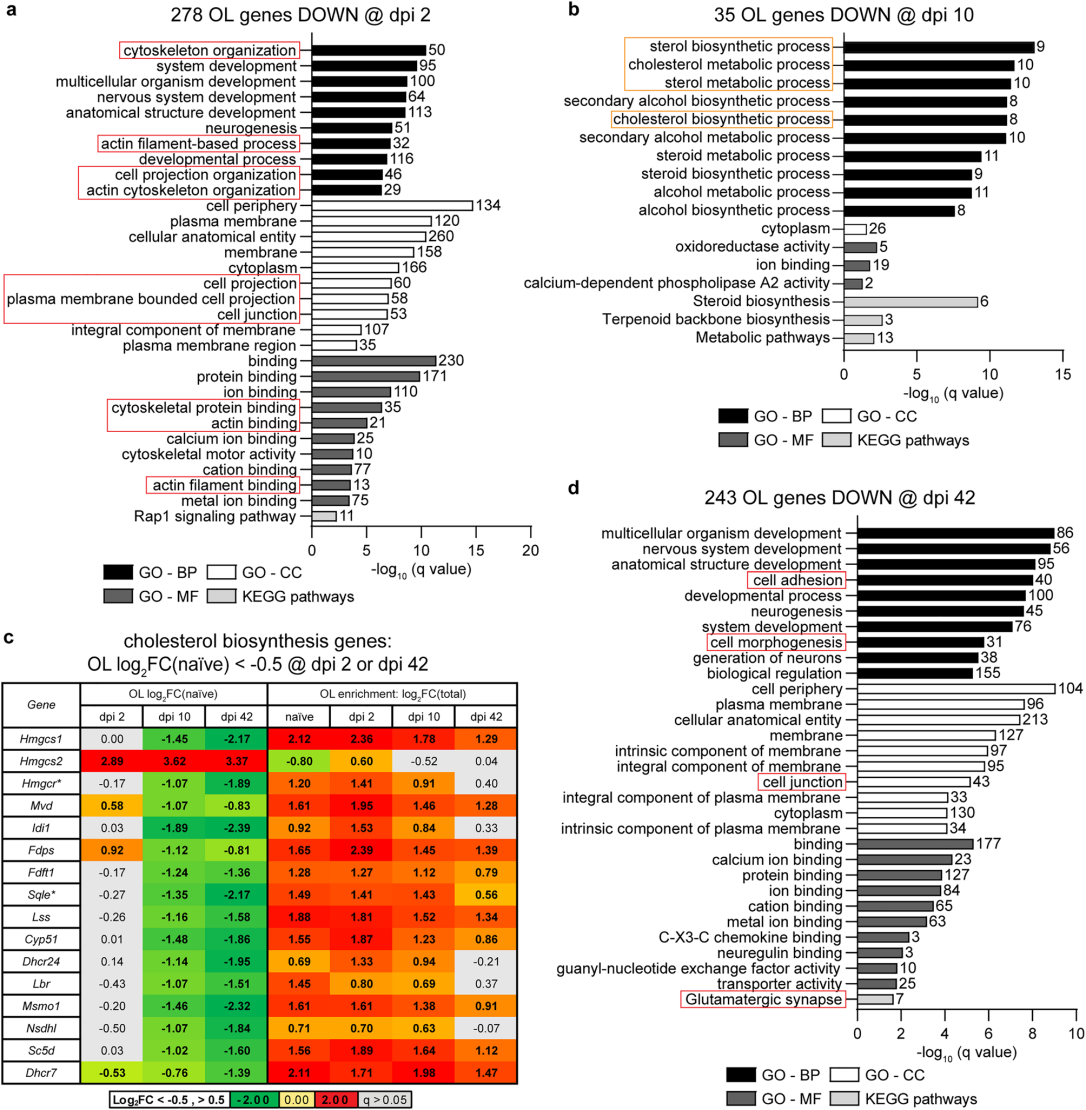
The OL response to SCI includes time-dependent downregulation of genes associated with morphological complexity, cell junctions, and cholesterol biosynthesis. (**a**,**b**,**d**) Overrepresented GOs (top 10/category) among high confidence OL translatome mRNAs that are highly downregulated after SCI (Log_2_FC/naïve/ < − 1, q < 0.05, and two-arm filtration /Fig. 3c/). On dpi 2 and 42, GOs associated with morphological complexity and cell junctions/cell adhesion are overrepresented (red boxes). On dpi 10, the top overrepresented GOs are related to cholesterol biosynthesis (orange boxes). (**c**) Fifteen out of 23 cholesterol biosynthesis genes that are OL-expressed are also OL downregulated on dpi 10 and 42 (Log_2_FC/naïve/ < − 0.5, q < 0.05, bold). All those downregulated genes are OL-enriched in naïve, dpi 2 and dpi 10 animals (Log_2_FC/Total/ > 0.5, q < 0.05, bold). For several of them, OL enrichment is lost by dpi 42 (q > 0.05, grey cells). Genes whose products are critical regulators of cholesterol biosynthesis are indicated (*). The chart of the cholesterol biosynthesis pathway with post-SCI changes in OLs is shown in Supplementary Fig. S2.

On dpi 2, 278 highly downregulated mRNAs from the OL-enriched translatome showed high level enrichment for several broadly defined GO-BP terms that are associated with development (Fig. 5a and Supplementary Table S4). In addition, several top enriched GO-BP, GO-CC, and GO-MF terms were related to cytoskeleton organization, cell projection organization, cell periphery, cell projections, cell junctions and actin cytoskeleton. While GO-BP cell adhesion (GO:0007155) was not among top enriched terms, 40 out of 271 genes in that category were also down with Log_2_FC(naïve) < − 1, − log(q) = 5.06). Such a functional profile of downregulated genes suggests adaptive changes to reduced morphological complexity of OLs and/or OL disconnection from other cells/extracellular matrix. Myelin sheath loss and disconnection from axons may be major drivers of these changes.

Although only 35 genes were highly downregulated in OLs on dpi 10, they showed significant overrepresentation of GO terms related to sterol biosynthesis (Fig. 5b and Supplementary Table S4). When data for all 23 OL-expressed components of the cholesterol biosynthesis superpathway were analyzed, 15 genes were downregulated (median Log_2_FC(naïve) = − 1.14, q < 0.05) and just one (*Hmgcs2*) was upregulated (Fig. 5c, Supplementary Fig. S2). Importantly, downregulated genes included two critical regulators of cholesterol biosynthesis *Hmgcr* and *Sqle* (Fig. 5c, Supplementary Fig. S2). Both genes remained downregulated on dpi 42 (Fig. 5c). Importantly, all downregulated cholesterol biosynthesis genes showed OL-enriched expression in naïve, dpi 2, or dpi 10 mice with median log_2_FC(Total) = 1.55, 1.61, or 1.39, respectively (q < 0.05, Fig. 5c). Such an expression pattern is consistent with a critical role of cholesterol synthesis in long term maintenance of myelin and survival of mature OLs^27^. Therefore, downregulation of cholesterol biosynthesis may represent an adaptive response to loss of myelinated axons/myelin sheaths acutely post-SCI. It may also contribute to OL apoptosis subacutely post-SCI^4,5^.

Top-enriched GOs for 224 transcripts that were highly downregulated on dpi 42 included several broadly defined terms such as development, biological regulation or binding (Fig. 5d and Supplementary Table S4). In addition, cell adhesion, cell junction and glutamatergic synapse were also enriched (Fig. 5d and Supplementary Table S4). This cell disconnection-like response resembles that observed on dpi 2. Collectively, GO analysis of OL-specific gene expression suggests a bioenergetic shift towards oxidative phosphorylation and reduction of morphological complexity/cell connectivity both acutely and subchronically post-SCI. At least subchronically, stress adaptation and OL survival is the likely outcome of such changes as most OL loss occurs during acute/subacute phases of the SCI recovery^4,5^. However, those surviving OLs appear to undergo protracted degeneration including putative disconnection from axons.

### Identifying candidate regulators of acute OL loss

**Figure 6:**
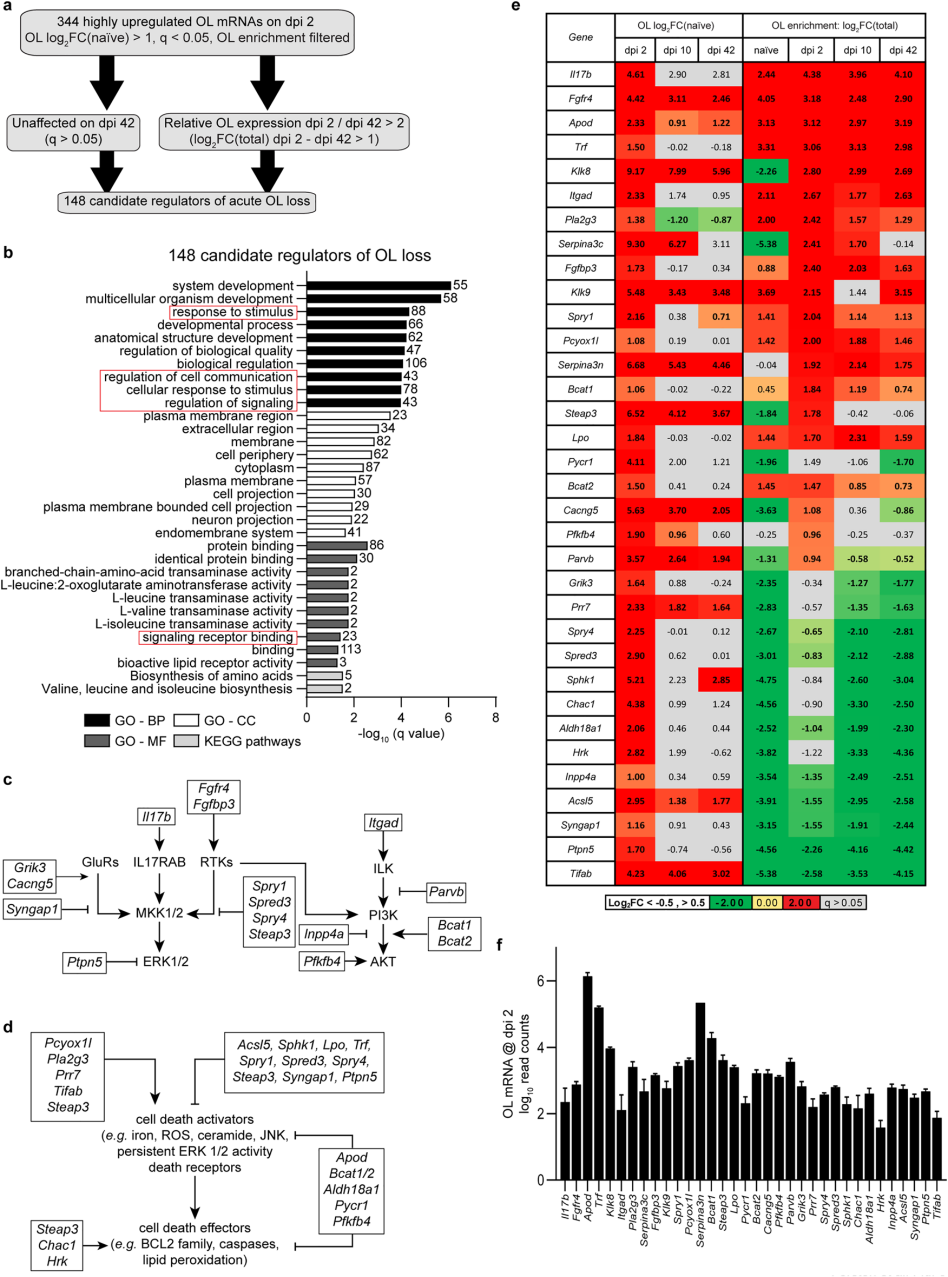
Identifying candidate regulators of OL loss after SCI. (**a**) To identify likely candidate gene regulators of OL loss, OL translatome mRNAs that were highly upregulated on dpi 2 were further analyzed to identify those whose expression normalized or declined at least twofold on dpi 42 when OL numbers stabilize and when little OL death has been reported (see text for details). (**b**) Overrepresented GOs (top 10/category) among candidate gene regulators of OL loss include several GO terms that are related to signaling (red boxes). (**c**,**d**) Literature-based analysis of candidate genes identified upregulated regulatory components of the survival signaling pathways including ERK and PI3K-AKT (**c**) as well as cell death activators and effectors (**d**). OL-upregulated genes are marked by black boxes. (**e**) OL translatome expression and OL enrichment of potential OL loss regulators that are listed in (**c**,**d**). Their dpi 2 read counts are shown in (**f**). In (**e**), genes with SCI-associated expression change or OL enrichment/depletion |Log_2_FC|> 0.5 are in bold (q < 0.05); non-significant effects are marked by grey cells (q > 0.05). In (**f**), log_10_ averages ± SD are shown. The full list of candidate genes and detailed results of GO analysis are in the Supplementary Table S5.

After SCI, most OL loss occurs at the injury epicenter 24–48 h post SCI^4,5^. Subacutely (dpi 7-dpi 21), additional OL loss via apoptosis is found in areas rostral and caudal from the epicenter^4,5^. No major OL loss has been reported beyond dpi 28^4,5^. Thus, OL mRNAs with acute post-SCI upregulation on dpi 2, but decreasing expression on dpi 42, may include major regulators of SCI-associated OL loss. Of 344 highly upregulated OL translatome mRNAs on dpi 2, 148 were either not significantly upregulated on dpi 42, or their dpi 42 levels were at least two-fold lower than on dpi 2 (Fig. 6a, Supplementary Table S5). GO analysis of those OL loss-associated mRNAs revealed enrichment for GO-BP terms related to cell signaling (Fig. 6b, Supplementary Table S5). Those included response to stimulus, regulation of cell communication, regulation of signaling, and signal transduction. Cell death-associated GO-BP terms were also moderately enriched (e.g. GO:1901214, regulation of neuron death, 12/381 genes, − log(q) = 2.80, Supplementary Table S5).

Literature analysis was performed to identify which of 148 acute-phase-specific mRNAs are likely regulators of OL loss. Interestingly, several of those transcripts encoded components of the two major OL survival signaling cascades: the ERK1/2 MAP kinase pathway and the PI3K-AKT pathway^28–30^ (Fig. 6c,e,f). Both positive and negative regulators of those pathways were upregulated (Fig. 6c). In case of the ERK1/2 MAP kinase pathway, increased activity of the glutamate receptor (*Grik3*, *Cacng5*)^31^ or receptor tyrosine kinase-mediated inputs (*Fgfr4*, *Fgfbp3*)^32^ appeared to be opposed by upregulation of negative regulators of RAS/RAC1-mediated activation of MKK1/2 (*Syngap1*^33^*, Spry1, Spred3, Spry4*^34^*, Steap3*^35^) or the ERK1/2 phosphatase *Ptpn5*^36^. In the case of the PI3K-AKT pathway, enhanced inputs (*Fgfr4*, *Fgfbp3*, *Itgad*, *Bcat1/Bcat2*, *Pfkfb4*)^37–39^ were opposed by upregulation of negative regulators including *Parvb*^40^ and *Inpp4a*^41^. Interestingly, OL upregulation of the pro-survival cytokine *Il17b*^42^ may represent an autocrine/paracrine stress survival mechanism as the IL17b receptor mRNA (*Il17rb)* is highly OL-enriched both in uninjured and contused spinal cord (Log_2_FC(Total) = 2.40, 2.87, 2.34, or 2.51 for naïve, dpi 2, 10, or 42, respectively, q < 0.05). Likewise, OLs appear as the main source of *Il17b* expression (Fig. 6e). Thus, pro-survival signaling networks are re-organized during the acute phase of OL response to SCI. Several of those changes may be adaptive and support survival under stress (*e.g*. activation of the ERK1/2-NFkB signaling by IL17B^42^, AKT activation by PFKFB4/BCAT1/2-mTOR^39,43^, or PTPN5-mediated dephosphorylation of the activated ERK1/2 to limit ERK1/2 signaling duration^44^).

Several acutely upregulated genes were identified as potential regulators of the cell death machinery (Fig. 6d–f). Activation of cell death may be promoted by: (i) reactive oxygen species (ROS) generating enzymes *Pcyox1l* and *Pla2g3*^45,46^, (ii) an inhibitor of the death receptor-driven, pro-survival gene transcription, *Tifab*^47^, (iii) a pro-excitotoxic inhibitor of JUN degradation, *Prr7*^48^ and/or (iv) a positive regulator of pro-apoptotic MAP kinases JNK/p38, *Steap3*^49^. Conversely, cell death initiation may be negatively regulated by increased expression of (*i*) enzymes that antagonize accumulation of the pro-apoptotic second messenger ceramide (*Acsl5*, *Sphk1*^50,51^), (ii) *Sphk1* which stimulates production of the cytoprotective lipid mediator sphingosine-1-phosphate^51^, (iii) negative regulators of ERK1/2 that may limit persistent, pro-necrotic activation of the ERK1/2 pathway in oxidative stress-exposed cells^44^, (iv) several enzymes that contribute to anti-oxidant defenses reducing ROS toxicity (*Lpo*^52^, *Apod*^53^, *Bcat1/2*^54^, *Aldh18a1*^55^, *Pyccr1*^56^, *Pfkfb4*^57^) and (v) reduced ROS generation due to *Trf* (transferrin)-mediated chelation of iron^58^. Likewise, the lipid-peroxidation mediated effector phase of cell death cascades including ferroptosis may be antagonized by those positive regulators of anti-oxidant defenses. Opposite effects on oxidative mechanisms of cell death execution are expected from upregulation of the potentially pro-ferroptotic lysosomal/endosomal ferroreductase *Steap3* which may promote lipid peroxidation^59–61^. Similar consequences may follow upregulation of the GSH-depleting enzyme *Chac1* compromising GSH-dependent lipid repair^61^. In addition, upregulation of the pro-apoptotic gene *Hrk* may promote SCI-induced OL apoptosis^62^.

Several upregulated genes with a potential to modulate oxidative cell death (*Pla2g3*, *Apod*, *Trf*, *Lpo*) encode for secreted proteins^63^. In addition, on dpi 2, those transcripts are highly OL-enriched, suggesting OLs to be the main source of their respective protein products in the contused spinal cord tissue acutely post-SCI (Fig. 6e). Other acutely OL-upregulated genes showing a similar pattern include those for secreted serine proteases (*Klk8*, *Klk9*) and secreted serine protease inhibitors (*Serpina3n*, *Serpina3c*, Fig. 6e). Excessive extracellular serine protease activity may promote cell death and enhance white matter damage^8,64,65^. Therefore, OLs appear to activate autocrine/paracrine mechanisms that may modify acute pathogenesis of SCI via regulation of oxidative stress and/or extracellular proteolysis. Such a concept is supported by a report that SCI-associated OL death and axonal damage was reduced in *Klk8*^*–/–*^ mice^8^.

### Epigenetic de-silencing as a potential regulator of the OL response to SCI

**Figure 7:**
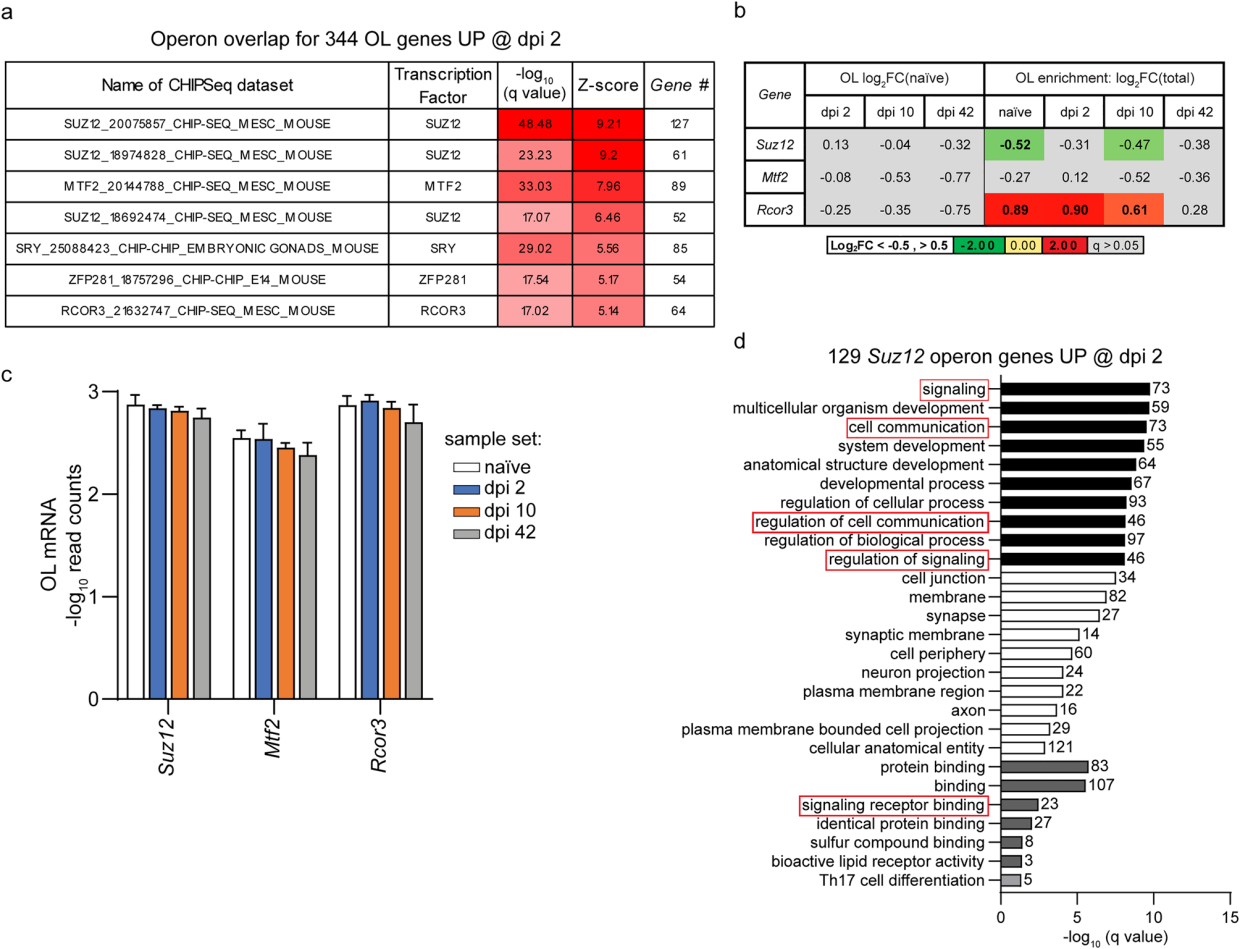
Epigenetic de-silencing as a potential regulator of the OL gene expression response to SCI. (**a**) Overlaps between 344 OL translatome mRNAs that are highly upregulated on dpi 2 and CHIP-Seq-confirmed mouse operon genes from publicly available data sets. Five of seven overlaps with z-scores > 5 are for operons of epigenetic regulators including SUZ12, MTF2, and RCOR3. A similar pattern of top overlaps was observed on dpi 10 and dpi 42 (Supplementary Table S6). (**b**,**c**) Relative (Log_2_FC) or absolute (Log_10_Read Count) expression of *Suz12, Mtf2, and Rcor3* in the OLs. In (**c**), data represent averages ± SD. (**d**) GO analysis of 129 targets of SUZ12 targets that are highly OL upregulated on dpi 2 (top 10 enriched GOs/category). Several GO terms related to cell signaling are also enriched (see Supplementary Table S6 for more data).

To identify mechanisms that may contribute to SCI-associated OL gene expression changes, overlaps between OL-upregulated genes and public datasets of ChIPSeq-confirmed mouse genome operons were determined using the ChIPSeq database module of the X2K suite^66^. At each post-SCI timepoint, top enriched operons included SUZ12 and MTF2, two components of the PRC2 chromatin repressive complex (Supplementary Table S6)^67^. To further validate the specificity of these enrichments, z-scores were calculated for operon overlap gene counts of 344 dpi 2 upregulated genes vs. average overlap gene count for 10 random sets of 344 OL expressed genes. OL-upregulated genes showed overlaps with seven mouse genome operons that passed a stringent specificity criterion of z > 5 (Fig. 7a). Four of those operons (including three with top z-scores) were for the chromatin silencing factor SUZ12. Two other chromatin silencing factors including RCOR3 and MTF2 were also highly enriched. The specificity of those findings was further confirmed by weak operon enrichment among 289 acutely downregulated genes with a z-score range for top ten overlaps of 0.94–2.57 (Supplementary Table S6).

SUZ12/PRC2-mediated repressive methylation of histones is required for OL differentiation by downregulating differentiation inhibitory genes^68^. RCOR3 is a negative regulator of the KDM1A-RCOR1/2/3-HDAC-driven gene silencing resulting in upregulation of genes that are repressed during cell differentiation^69^. *Suz12*, *Mtf2,* and *Rcor3* transcripts were detected both in total RNA and OL translatomes (Fig. 7b,c). Their expression was unaffected by SCI. While *Suz12* and *Mtf2* showed similar expression in OLs and total RNA, *Rcor3* was moderately OL-enriched in all groups except dpi 42 (Fig. 7b,c). Therefore, the identified operons are matched by continuous OL expression of their respective regulators.

Interestingly, the list of 129 unique SUZ12 target genes that were OL-upregulated acutely after SCI showed overrepresentation of GO terms related to signaling (Fig. 7d) and included several potential regulators of acute OL loss (Supplementary Table S6). Most those PRC2 targets overlapped with the RCOR3 operon (Supplementary Table S6). The current data suggest that de-silencing of the PRC2 and RCOR3 operons may contribute to acute upregulation of many SCI-response genes in OLs. Moreover, de-silencing of PRC2-repressed genes may also play a role in OL gene upregulation on dpi 10 and dpi 42.

### STEAP3 as a novel marker of acute OL response to SCI

STEAP3 is a membrane protein that acts as a major endosomal/lysosomal metalloreductase that reduces Fe^3+^/Cu^3+^ to Fe^2+^/Cu^2+^^59,70^. As Fe^2+^ is then exported from endosomes/lysosomes to cytoplasm, STEAP3 plays an important role in the cellular iron supply^59,60,71^, but may also promote lipid peroxidation and ferroptosis^72^. In addition, STEAP3 binds to cell death effectors and signaling mediators to promote apoptosis and/or reduce activity of the pro-survival pathways^35,49,73^. It is also required for stress-dependent exosome secretion^74,75^. Therefore, OL-upregulated *Steap3* may promote OL loss, but may also, via stimulation of exosome-mediated secretion, affect axons and/or other spinal cord cells.

**Figure 8:**
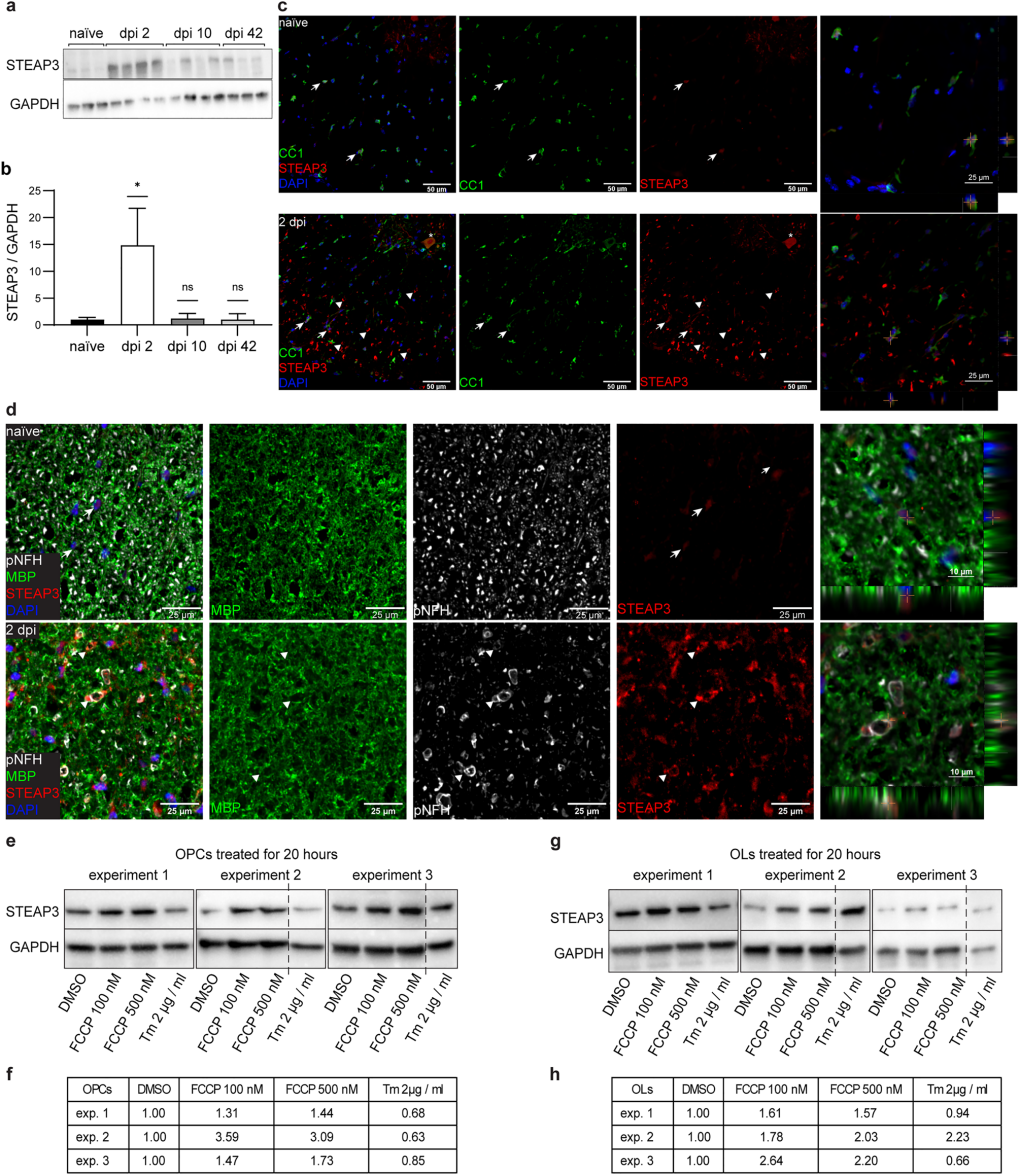
STEAP3, a candidate regulator of OL loss, is upregulated acutely after SCI. (**a**) A representative western blot using protein lysates from the contused spinal cord tissue of WT mice at the indicated post-injury times. Each lane corresponds to an individual mouse. (**b**) Quantification of the blot shown in (**a**). GAPDH-normalized STEAP3 expression is significantly elevated on dpi 2 (means ± SD; *p < 0.05; ns, p > 0.05 Mann–Whitney *u*-test). (**c**,**d**) Representative confocal images of co-immunostainings for STEAP3 and the markers of OL cell somas (CC1), myelin sheaths (MBP) or axons (p-NFH) in the ventral white matter from WT mice with (dpi 2) or without SCI (naïve). Cell nuclei were counterstained with Hoechst-33258 (DAPI). The SCI sections come from a region 1–2 mm rostral from the injury epicenter. (**c**) Arrows indicate CC1^+^ OLs that show weak STEAP3 signal. Such cells are observed both in control tissue and on dpi 2. Arrowheads mark ring-like structures with a strong STEAP3 signal that do not overlap with cell nuclei and are only observed after SCI. A STEAP3^+^ neuron-like cell in the grey matter is indicated by an asterisk. (**d**) Arrows indicate cells with STEAP3 staining. Arrowheads identify damaged, distended axons that are associated with a strong STEAP3 signal. Similar STEAP3^+^ structures were also observed on dpi 1 (Supplementary Fig. S4). Staining specificity controls are shown in Supplementary Figs. S3 and S4. (**e**–**h**) Cultured rat OPCs or OPC-derived OLs were treated as indicated. GAPDH-normalized STEAP3 expression was analyzed by western blotting (**e**,**g**) and quantified as fold of vehicle (DMSO)-treated controls (**f**,**h**). Increased STEAP3 expression with the mitochondrial uncoupler FCCP but not the ER stress inducer tunicamycin (Tm) was replicated in 3 independent experiments. Dotted lines (**e**,**g**) indicate deletion of lanes with samples that were unrelated to the current study. Original blots are presented in the Supplementary Fig. S5.

In OLs, *Steap3* was sharply up on dpi 2 and remained elevated, albeit at reduced levels throughout the recovery period with Log_2_FC(naive) ranging from 6.52 on dpi 2 to 3.67 on dpi 42 (Fig. 6e). In total RNA samples, *Steap3* levels were also increased throughout the recovery period with an apparent peak on dpi 2 (Log_2_FC(naive) = 2.9, 2.7, or 1.89 on dpi 2, 10, or 42, respectively, q < 0.05). On dpi 2, *Steap3* was highly OL-enriched (Fig. 6e). At that timepoint, increased expression of STEAP3 protein was also confirmed by western blotting (Fig. 8a, b, Supplementary Fig. S5a). Therefore, at least acute OL translatome upregulation of *Steap3* transcript correlates with increased bulk spinal cord expression of STEAP3 protein.

In naïve mice, faint STEAP3 immunofluorescence was observed in cell bodies of white matter OLs (CC1^+^, Fig. 8c, Supplementary Fig. S3). On dpi 1 or 2, such staining was also present throughout the spared ventral white matter at the injury epicenter as well as rostrally or caudally (Fig. 8c, Supplementary Figs. S3 and S4). Seemingly nuclear localization of OL STEAP3 staining on transverse sections may represent perinuclear localization that is polarized mainly in the rostral-caudal axis (Fig. 8c). Unipolar, perinuclear STEAP3 immunofluorescence that reflects localizations to the endosomal/trans-Golgi network (TGN) has been reported in various cell types^59,70,74,75^.

Acutely after SCI (dpi 1 or 2), a strong STEAP3 signal was also found in ring-like structures throughout the spared white matter (Fig. 8c,d, Supplementary Figs. S3 and S4). Those structures were observed both at the injury epicenter as well as at the penumbra region rostrally and caudally from the injury site. They often showed partially overlapping and/or closely associated signals for the myelin marker MBP, as well as axonal marker phospho-NFH (p-NFH, Fig. 8d, Supplementary Fig. S4). Such close associations without a complete overlap suggest that after SCI STEAP3 marks OL paranodes and/or a peri-axonal compartment adjacent to damaged axons. However, testing such possibilities will require future imaging studies using longitudinal sections. Importantly, the STEAP3^+^ structures likely represent a specific STEAP3 signal, as they were not observed with a control IgG (Supplementary Figs. S3 and S4). Therefore, acute upregulation of OL STEAP3 may be directly related to degeneration of myelinated axons.

Interestingly, STEAP3 was upregulated following treatment of cultured OL precursor cells (OPCs) or OPC-derived OLs with a mitochondrial uncoupling drug FCCP, but not the ER stress inducer tunicamycin (Fig. 8e–h, Supplementary Fig. S5b,c). As FCCP uncouples mitochondrial respiration from ATP synthesis and increases mitochondrial ROS generation^76^, upregulation of STEAP3 may represent a response of OL lineage cells to mitochondria dysfunction/mitochondrial oxidative stress. The latter form of cellular damage may upregulate expression of iron supply genes as mitochondrial biogenesis is a major driver of iron demand^77^.

Finally, OL expression of another acutely upregulated and OL-enriched candidate pro-oxidant transcript, *Pcyox1l*^45^ was confirmed by immunostaining. Unlike STEAP3, the PCYOX1L signal was observed mainly in cell bodies and adjacent processes of CC1^+^ or CNP^+^ OLs of the ventral white matter (Supplementary Fig. S6). Therefore, OLs may directly contribute to SCI-associated oxidative stress.

## Discussion

The current data set represents the first comprehensive transcriptomic/translatomic description of OL gene expression response to SCI. Acutely, SCI-challenged OLs show translatomic changes indicative of a metabolic shift towards mitochondrial respiration that coincides with morphological simplification/cellular disconnection. Unexpectedly, a similar pattern reemerges subchronically. Acutely, OLs appear to undergo extensive re-organization of survival signaling networks. In addition, the acute OL translatome changes suggest an active role in regulation of cytotoxic mechanisms that contribute to secondary injury including extracellular proteolysis and oxidative stress. Epigenetic de-silencing appears to be a major driver of the SCI-activated OL gene expression. Lastly, STEAP3 is a novel marker of OL injury response that may play a dual role as a positive, pleiotropic regulator of OL death/degeneration and an enhancer of exosome-mediated intercellular communication.

The current translatome analysis revealed OL-specific, SCI-mediated upregulation of several genes that were also identified as OL-upregulated in a recent scRNASeq analysis of the mouse spinal cord tissue acutely/subacutely after moderate contusive SCI^10^. Those include *Apod*, *Pla2g3*, *Trf*, *Klk8*, *Serpina3c*, *Serpina3n*, *Steap3*, *Pcyox1l*, *Itgad*, *Pfkfb4*, *Bact1/2* or *Spry1*. In addition, SCI-associated OL translatome changes show partial overlap with OL transcriptomic response to other types of CNS injury^78^. Recent analysis of multiple scRNASeq datasets from various mouse models of white matter damage including amyloidosis, tauopathy, and cuprizone or lysolecithin-induced demyelination has identified three major transcriptomic profiles of disease-associated OLs called DA1, DA2 and IFN^78^. Of note, signature transcripts of all those profiles showed significant overlaps with SCI-upregulated OL mRNAs (Supplementary Fig. S7). The greatest overlaps were observed for the persistent, pro-inflammatory DA1 cluster. The overlap peaked on dpi 10, but was clearly present also on dpi 2 and dpi 42. Interestingly, *Steap3* has been identified as a marker of DA1^78^. The overlap with the transient cell injury/cell survival response profile DA2 peaked on dpi 2. At that time, a strong overrepresentation of the inflammation-response cluster IFN was also present. In addition, similar to subacute and subchronic OL SCI translatomes, all disease-associated OL gene expression profiles showed downregulation of cholesterol biosynthesis^78^.

While the current OL translatome dataset captures a large set of OL-expressed genes including those that are regulated by injury, it has important limitations that should be considered when choosing analysis tools or interpreting data. First, we observed apparent contamination of OL polysomes with mRNAs from non-OL cells including microglia/macrophages. Such a contamination may originate from non-specific interactions between highly abundant non-OL mRNAs and IgG-coupled magnetic beads that occurs during sample preparation. Similar contamination has been recently reported in co-cultures of human and mouse neural cells^25^. The two-fold filtration process that took into account differential or constantly high OL enrichment reduced that contamination and identified high confidence components of the OL translatome that are regulated by SCI. However, while specificity of detection has improved, sensitivity likely suffered with over 60% of differentially expressed OL translatome mRNAs not passing the filtration criteria (Fig. 3). Therefore, some components of OL response are likely missed in our analysis. Nevertheless, such a conservative analysis approach is justified in acute CNS injury models where dramatic changes in both tissue cellular composition and transcriptomes of inflammatory cells are major factors that determine bulk transcriptome readouts.

Another limitation is related to imperfect OL specificity of *Plp*-*CreERT2* expression. The *Plp-CreERT2* transgenic line that was used here to activate the Ribotag also drives Cre-ERT2 activity in Schwann cells^79^. As spinal cord samples my contain some short fragments of spinal nerve roots, it is possible that a small fraction of OL polysomes is of Schwann cell origin. Such a contamination of the current dataset is suggested by an apparent OL enrichment and SCI upregulation of marker transcripts of Schwann cell nerve injury response including *Egfl8* or *Gdnf* (Supplementary Table S2)^80^. Hence, at least some SCI-associated effects on OL gene expression may represent Schwann cell responses. Potential classification mistakes can be best avoided by confirming OL translatome mRNA changes using single cell level analysis of protein expression such as immunofluorescence or in situ hybridization-based approaches.

Last, but not least, the current study was limited to female mice, potentially missing male-specific components of the OL SCI translatome. As more post-operative complications arise after experimental SCI in males compared to females, including difficulty in bladder expression and increased frequency in urinary infections, the majority of rodent SCI literature uses females. Selecting only females for experimental SCI therefore provides a more successful survival rate and reliable sample size. For instance, several cell type specific SCI gene expression datasets were obtained from C57Bl6 female mice^10,17,18^. Importantly, in that genetic background, post-contusion tissue loss/locomotor recovery shows subtle, mostly non-significant differences between males and females^81,82^. Therefore, the current SCI dataset from C57Bl6 OL-Ribotag females may miss only few components of the OL translatome that are biologically relevant for tissue sparing and/or recovery in males.

In myelinating glia, mitochondrial respiration is critical for myelination during development as myelin synthesis requires a large amount of ATP^83,84^. Conversely, in mature OLs, mitochondrial respiration is dispensable for OL survival, myelin sheath maintenance and axonal function^83^. Instead, OLs support axonal energy needs by providing lactate that is generated via aerobic glycolysis^83^. As electrical activity of axons is a major consumer of OL-generated lactate^83^, one could expect that OL disconnection from axons and/or axonal damage would lower lactate demand. Consequently, aerobic glycolysis would be reduced and mitochondrial respiration would increase. Our observed concomitant upregulation of respirasome genes, together with downregulation of genes associated with morphological complexity/connectivity, supports such a scenario both acutely and subchronically after SCI. Indeed, while most SCI-associated axonal loss is acute, continuing subchronic degeneration of myelinated axons has been described^85,86^. In addition, subchronic reorganization of OL respiration may be a response to post-SCI reduction in activity of specific axonal tracts and/or spinal circuitries.

Interestingly, SCI-mediated changes in OL gene expression indicate their active role in modifying the tissue environment to modulate secondary injury. Such effects may be mediated both by canonical secretion of signal-peptide containing proteins and non-canonical secretion by exosomes, as STEAP3 is a major regulator of exosome-mediated secretion^74,75^. Of note, significant enrichment of secreted proteins among upregulated genes on dpi 10 or dpi 42 suggests that OLs regulate not only acute damage, but also subacute/subchronic repair of the contused spinal cord.

In summary, this study establishes the first translatomic chart of OL response to thoracic contusive SCI. Acutely upregulated candidate regulators of OL survival are identified. Their increased expression is likely driven by axonal disconnection, oxidative damage, and inflammation. Subchronic gene expression changes suggest reduced myelination and decreased axonal trophic support by surviving OLs. Moreover, upregulation of various secretome genes and transcriptional similarity to immunogenic OLs from other white matter injury models^78^ (Supplementary Fig. S7) supports an active role of OLs in tissue damage and repair throughout the post-SCI recovery period. Importantly, previously unrecognized aspects of OL biology after SCI are uncovered. Those include putative metabolic re-programming, re-organization of intracellular signaling and epigenetic de-silencing as a major driver of OL gene expression response to injury. At least some of those newly identified elements of SCI pathogenesis may be further validated as potential targets for therapies to reduce white matter damage and/or dysfunction after SCI. For instance, reducing oxidative phosphorylation in OLs or targeting OL upregulated cell death genes may attenuate acute loss of OLs. Subchronically, therapeutic downregulation of oxidative phosphorylation or upregulation of cholesterol biosynthesis may be beneficial for long-term maintenance of white matter integrity and/or axonal function. Finally, the SCI-associated OL translatome data will be useful for design and/or interpretation of mechanistic studies of SCI-associated white matter damage as well as other types of white matter pathology. Current data are available as a searchable database at SCI OL Gene Expression Database (scigenedatabase.com).

## Materials and methods

### Animals

All animal procedures were performed in strict accordance with the Public Health Service Policy on Humane Care and Use of Laboratory Animals, *Guide for the Care and Use of Laboratory Animals* (Institute of Laboratory Animal Resources, National Research Council, 2011), and adhered to NIH guidelines on the use of experimental animals. Animal procedures were approved by the University of Louisville Institutional Animal Care and Use (IACUC) and Institutional Biosafety (IBC) Committees. Timed-pregnant Sprague–Dawley rats and wild-type (WT) C57Bl/6 mice (6–8 weeks) were obtained from Envigo (Indianapolis, IN). *Plp-cre*^*ERT2*^ (proteolipid protein) (B6.Cg-Tg (Plp1-Cre/ERT)3Pop/J; Stock No: 005975)^79^ and Ribotag mice (*Rpl22*^*fl(STOP)fl-HA/wt*^, B6J.129(Cg)-Rpl22 ^tm1.1Psam2^/SjJ); Stock No: 029977)^16^ mice, both on C57Bl/6 background, were acquired from the Jackson Laboratory (Bar Harbor, ME). These lines were crossed to produce OL-Ribotag mice (*Plp1-Cre*^*ERT2*+*/wt*^*:Rpl22*^*fl(STOP)fl-HA/wt*^). Genotypes of the crosses were confirmed using standard PCR genotyping as recommended by the Jackson Laboratory. To induce OL Ribotag expression, 6 week old OL-Ribotag mice received 1 mg tamoxifen (20 mg/mL in sunflower oil) i.p. daily for 8 days as previously described^87^. Male and female mice were used for initial validation of Ribotag transgene induction. Reporting of animal studies described in this manuscript followed ARRIVE guidelines.

### SCI

Female WT or OL-Ribotag mice were used for SCI at 8–10 weeks of age. In tamoxifen-induced mice, SCI was performed 3 weeks after completion of the induction treatment. Anesthetized animals (400 mg/kg body weight 2,2,2-tribromoethanol i.p.) were shaved around the surgical site and disinfected using 4% chlorohexidine solution. Lacri-Lube ophthalmic ointment (Allergen, Madison, NJ) was applied to prevent eye drying. Following dorsal laminectomy at the T9 vertebrae, moderate contusive SCI was performed using the IH impactor (50 kdyn force/400–600 μm displacement, Infinite Horizons, Lexington, KY) as previously described^88^. Starting immediately after surgery, postoperative care included 0.1 ml saline (s.c. daily for 7 days), 5 mg/kg gentamycin (s.c. daily for 7 days), 0.1 mg/kg buprenorphine (s.c. every 12 h for 2 days), and manual expression of bladders twice a day for seven to ten days or until spontaneous voiding returned. All surgical and post-surgery procedures were completed according to NIH and IACUC guidelines. All surgeries were performed without knowledge of group assignment or genotype.

### Tissue collection

Anesthetized mice were transcardially perfused with phosphate buffered saline (PBS, 4 °C). For immunostaining, this was followed by 4% paraformaldehyde (PFA in PBS, 4 °C) perfusion. Then, a 5 mm portion of the spinal cord spanning the injury epicenter was dissected and (i) post-fixed for 1 h in 4% PFA at 4 °C (immunostaining) or (ii) flash frozen in liquid nitrogen and stored at − 80 °C until further use (Ribotag polysome purification or RNA/protein isolation).

### Immunostaining

Tissue processing, preparation of frozen 20 μm coronal (transverse) spinal cord sections, immunostaining and epifluorescent (RPL22-HA) or confocal (STEAP3, PCYOX1L) imaging followed standard protocols as previously described^88,89^ (see Supplementary Methods for more details). Primary antibodies used were as follows: anti-APC (mouse CC1 clone, 1:200, Abcam, Cambridge, UK, Cat# ab16794, RRID: AB_443473), anti-HA (mouse, 1:1000, HA.11 Clone 1612, Biolegend, San Diego, CA, Cat# 901516, RRID: AB_2820200), anti-STEAP3 (species: rabbit, dilution: 1:200, Thermo Fisher Scientific, Walthanm, MA, Cat# PA5-102321, RRID: AB_2851729), anti-phospho-NFH (species: mouse, dilution: 1:500, Biolegend Cat# 801601, RRID: AB_2564641), anti-MBP (species: chicken, dilution: 1:500, Thermo Fisher Scientific Cat# PA1-10008, RRID: AB_1077024), anti-PCYOX1L (species: rabbit, dilution: 1:200, Atlas Antibodies, Bromma, Sweden, Cat# HPA037463, RRID: AB_10673632), and anti-CNP (species: mouse, Clone SMI 91, dilution: 1:200, Biolegend Cat# 836404, RRID AB_2566639). Species- and isotype-specific Alexa488-, Alexa594-, or Alexa647- F(ab’)2 secondary antibodies (donkey, 1:200, Life Technologies) were used. Counting of Ribotag/HA^+^ cells was performed without knowledge of sample origin (genotype or tamoxifen treatment).

### Ribotag RNA purification and RNASeq

Frozen spinal cord samples from 2 female mice were pooled to produce one biological replicate (3 biological replicates/group representing 6 animals). Pooling was done to increase RNA yield of the Ribotag immune-purification as determined in pilot experiments. SCI and naïve mouse spinal cord samples were processed for input polysome-associated mRNAs (total spinal cord mRNA) or immune-purified OL polysome mRNAs using anti-HA antibody and magnetic beads as previously described^16,90^ (see Supplementary Methods for a detailed description of the protocol). RNA isolation, mRNA library preparation and RNA sequencing on the Illumina NextSeq 500 platform followed standard procedures. Before preparing RNASeq libraries, successful isolation of OL polysomes was verified by qPCR for OL- and non-OL cell marker transcripts. Pilot studies revealed that polysome immunoprecipitation using a control IgG produced low RNA yields as compared to the anti-HA antibody confirming the specificity of the latter reagent (average of 48.4 or 285.34 ng RNA/sample with IgG or anti-HA, respectively. Given such a disparity in RNA recovery between the control IgG and the anti-HA antibody, further analyses focused on anti-HA-purified OL translatomes.

### Quantitative real-time PCR

To prepare cDNA, the SuperScript IV system was used following manufacturer’s recommendations (Thermo Fisher, Cat# 18091050). qPCR was run using a microfluidic Custom TaqMan Gene Expression Array Card (Thermo Fisher, Cat# 43442249) containing primers for CNS cell-type specific marker mRNAs of oligodendrocytes (*Mbp, Plp1, Mog, Cldn11, Mobp, Opalin, Mag, Fa2h, Gjb1, Ermn, Gjc2, Klk6, Sox10*), astrocytes (*Aldh1l1, Hgf*), neurons (*Reln, Snap25, Lhx5*), and microglia/macrophages *(Osm, Cd68, Tmem19).* The card design is shown in Supplementary Methods. RNA levels were quantified using the ΔΔCT method with *Hprt, Ppia,* and 18S rRNA as reference transcripts. For each sample pair (OL translatome and total spinal cord RNA from which OL translatome was isolated), OL mRNA levels were determined as a fold change of their total spinal cord expression.

### RNAseq data analysis

Analysis of sequenced RNA was performed by the Kentucky IDeA Networks of Biomedical Research Excellence (KY INBRE) Bioinformatics Core. A quality control analysis was performed using FastQC (v.0.10.1) and indicated good sequencing quality. The reads were directly aligned to the *Mus musculus* reference genome (mm10.fa) using the STAR aligner (version 2.6). The average number of sequenced reads per sample was ~ 37,500,000 with an average alignment rate of 98.06%. Raw read counts were generated using HTSeq (v.0.10.0) and input to DESeq2 for differential expression analysis. The raw counts were normalized using Relative Log Expression (RLE) and filtered to exclude genes with fewer than 10 counts across samples. Principal component analysis (PCA) was performed on all 24 samples to measure variance of the overall mRNA expression pattern across all groups/sample sets. As part of the differential expression analysis, a DESeq2 interaction term was used to analyze differential OL enrichment after SCI and can be defined by delta Log_2_FC(OL/total) = Log_2_FC(OL/total)_SCI – Log_2_FC(OL/total)_naive. In this analysis, OL translatome- and total spinal cord RNA samples from the same tissue were paired for determination of differential OL enrichment. To identify genes with an RNA origin-specific effect (OL vs. total) at one or more time points, the full regression model was compared to a reduced model using a likelihood ratio test. Group-specific effects were then identified at individual timepoints using a Wald test for significance. As a significant contamination of non-OL transcripts has been detected in OL translatomes from SCI samples, a two arm filtration process was implemented to identify highly likely components of the OL translatome that are differentially expressed after SCI (Fig. 3a–c). In arm 1, the identified transcripts were filtered using the DESeq2 interaction function to identify those that also showed differential OL enrichment of the same direction/magnitude (change of OL enrichment in SCI vs. naïve samples, |Log_2_FC/Total/_SCI_-Log_2_FC/Total/_naïve_|> 1, q < 0.05). This process eliminated mRNAs whose OL translatome changes are driven primarily by their parallel changes in the total RNA pool. However, differential enrichment analysis may miss those mRNAs whose OL translatome levels change in the same direction/magnitude as in total spinal cord samples. In case of constantly OL-enriched mRNAs that also show preferential OL expression (Log_2_FC(Total) > 1, q < 0.05), their exclusion is not justified as OLs are their major expressors and cross-contamination from other cell types is less likely. In total RNA samples, injury related changes of such OL-enriched transcripts are still a reflection of OL-specific response to SCI. Therefore, the second arm of the filtration procedure identified those highly regulated mRNAs that were also OL enriched regardless of passing the differential enrichment filtration. All RNASeq data are available in GEO (Accession number: GSE225308). A searchable, public database is also available at http://scigenedatabase.com/.

Gene ontology (GO) functional annotation analysis was performed using g:Profiler (version *e*108_eg55_p17_d098162)^91^. Mitochondrial respirasome genes (Respirasome_GO_0005746) were retrieved from g:Profiler, and cholesterol biosynthesis superpathway genes were retrieved from Kegg Pathway (mmu:00100 and mmu:00900) and BioCyc databases^92^. Mitochondrial respirasome (Respirasome_GO_0005746) and cholesterol biosynthesis superpathway gene lists were retrieved from Metascape or Kegg Pathway (mmu:00100 and mmu:00900) databases, respectively.

#### Gene list overlap analyses

Lists of top 500 neural cell type-enriched transcripts were obtained from the BrainRNASeq database (www.brainrnaseq.org)^93^. Their overlaps with the SCI-regulated OL mRNAs were analyzed using the hypergeometric test (http://nemates.org/MA/progs/overlap_stats.html). In those calculations, the maximal number of all identified transcripts across all analyzed samples (17,168) was used to define the size of the whole spinal cord transcriptome.

### Immunoblotting

Immunoblotting followed standard methodology as previously described (see Supplementary methods for more details)^88^. The primary antibodies included STEAP3 and GAPDH (loading control) (Chemicon, 1:1000, Temecula, CA). BioRad ChemiDoc MP Imaging System was used. Immunoblot quantifications were performed using TIFF-formatted blot images and BioRad Image Lab software.

### Cell culture

Culture and OL differentiation of adult rat spinal cord OPCs followed previously described methodology^94^. Cells were treated as follows: FCCP (50 μM working stock solution in 0.1%DMSO/cell culture medium that was diluted to final concentrations 500 nM or 100 nM in cell culture medium; the FCCP stock was always prepared fresh), Tunicamycin (10 mg/ml stock prepared in DMSO and diluted to the final concentration of 2 μg/ml in culture medium) or their vehicle (0.02% DMSO in culture medium). Each solution was made fresh on day of treatment.

### Statistical analysis of immunostaining, qPCR and immunoblotting data

All ratiometric data including % Ribotag^+^ cells, qPCR-determined total spinal cord-normalized OL mRNA levels and control treatment-normalized protein expression were performed using the two-tailed non-parametric Mann–Whitney *u*-test.

### Supplementary Information

Supplementary Information.

Supplementary Table S1.

Supplementary Table S2.

Supplementary Table S3.

Supplementary Table S4.

Supplementary Table S5.

Supplementary Table S6.

## Data Availability

All key data are part of the manuscript and/or are available in the public repository (NCBI GEO Accession number: GSE225308) or via authors web page (http://scigenedatabase.com/). Additional data would be shared by the corresponding author upon reasonable request.
