# Supplementary material for: Enhanced oxidative phosphorylation, re-organized intracellular signaling, and epigenetic de-silencing as revealed by oligodendrocyte translatome analysis after contusive spinal cord injury
Source: Sci Rep. 2023 Dec 1;13:21254. doi: 10.1038/s41598-023-48425-6 (PMC10692148; doi:10.1038/s41598-023-48425-6)
Supplement: Supplementary file 1 — Supplementary Information. [file 41598_2023_48425_MOESM1_ESM.pdf]

Enhanced oxidative phosphorylation, re-organization of intracellular signaling, and epigenetic de-silencing as revealed by oligodendrocyte translatome analysis after contusive spinal cord injury.

Michael D. Forston, George Wei, Tyler Stephenson, Kariena Andres, Julia H. Chariker, Charles Glover, Eric C. Rouchka, Scott R Whittemore, Michal Hetman

<u>Supplementary Materials</u>

1. Supplementary Methods
2. Legends for Supplementary Figures S1-S6
3. Supplementary Figures S1-S6
4. List of Supplementary Tables S1-S6 (provided as separate excel files)

Supplementary Methods

<u>Ribotag translatome purification.</u> Frozen tissue samples were homogenized with pre-chilled Dounce homogenizer (40 strokes using type A pestle, 40 strokes using type B pestle) in cold homogenization buffer on ice [RNAse-free water (Invitrogen), 50 mM Tris (Sigma), pH 7.5, 100 mM KCl (Sigma), 12 mM $MgCl_2$ (Sigma), 1% Nonidet P-40 (Sigma), 200 U/mL RNAsin (Promega, catalog #N2115), 1 mM DTT (Sigma-Aldrich), proteinase inhibitors (Roche), 1 mg/mL heparin (Sigma-Aldrich), 0.1 mg/mL cyclohexamide (Sigma-Aldrich)] to a wt/vol ratio of ~2-5%. Due to low yields of RNA from OL samples with this procedure, 2 animal tissue samples were pooled together to produce one biological replicate (6 animals per time point, n = 3 biological replicates per time point). Total RNA samples were also pooled respective to the OL samples for consistency. Homogenates were centrifuged at 10,000 x g for 10 minutes at 4°C to remove excess tissue. 10% of the cleared lysate was taken as input (IN, total spinal cord RNA) and stored at -80°C for subsequent analysis. The remaining lysate was incubated with monoclonal mouse anti-hemagglutinin (HA) antibody (HA.11 Clone 1612, Biolegend, Cat# 901516, RRID: AB_2820200) at a concentration recommended by

Sanz et al. (2019) for 4-6 h at 4°C in a microtube rotator and end-over-end gentle mixing. Protein A/G magnetic beads (Pierce) were prewashed in homogenization buffer and added to the lysates and further incubated at 4°C overnight with end-over-end mixing. The following day, samples were placed in a magnetic microtube stand on ice to isolate the magnetic beads, and the supernatant was stored in -80°C. Magnetic beads with the bound immunoprecipitated mRNA (IP) were washed 3 times using ice-cold high salt buffer solution (50 mM Tris, pH 7.5, 300 mM KCl, 12 mM $MgCl_2$, 1% Nonidet P-40, 1 mM DTT, 100 g/mL cycloheximide) for at least 5 mins at 4°C and end-to-end mixing. Input and immunoprecipitated mRNA were purified using RNeasy Mini, RNA isolation kit (Qiagen, catalog # 74104) and Arcturus PicoPure RNA Isolation Kit (Applied Biosystems, catalog # LSKIT0204) according to the manufacturers' protocol, respectively. Concentration and quality of isolated RNA was first determined using a NanoDrop 1,000 spectrophotometer (Thermo Scientific). The concentration was then measured by Qubit Fluorometer (Thermo Scientific), and RNA integrity was assessed by capillary electrophoresis (Bioanalyzer, Agilent Technologies). Library preparation for single-end RNA sequencing was done using the Universal Plus mRNA-seq (NuGEN Cat #0508) and included Poly(A) selection to purify mRNA, RNA fragmentation, cDNA synthesis, cDNA purification, end repair, adaptor ligation, strand selection, strand selection purification, library amplification and library purification. Sequencing was performed on Illumina NextSeq 500 utilizing the NextSeq 500/550 75 cycle High Output Kit v2.5 (20024906).

RNASeq data analysis. Quality control (QC) was performed using FastQC (v.0.10.1) for each sequencing run. RNA sequencing produced ~900,000,000 reads across all 24 samples. The average number of reads per sample was ~37,500,000 and ranged from ~36,130,000-39,500,000 reads across samples. Reads were aligned to the *Mus musculus* reference genome (mm10) using STAR (v.2.6) with an alignment rate above 97% for all samples. The average number of aligned reads per sample was ~36,800,000 and ranged from ~35,500,000-38,700,00 reads across samples. Raw read counts were obtained using HTSeq (v.0.10.0) and were input to DESeq2 (v.1.24.0) for differential expression analysis. As part of the analysis, raw read counts were

normalized using the Relative Log Expression (RLE) method and filtered to exclude genes with fewer than 10 counts (reads) across samples.

Immunostaining. Tissue processing, preparation of frozen 20 µm coronal (transverse) spinal cord sections and immunostaining methodology followed standard protocols as previously described (Ohri et al., 2011) (Bankston et al., 2019)(see Supplementary Methods for more details). Post-fixed tissue was cryoprotected (30% sucrose, 72 h at 4°C), blocked in tissue freezing medium (Triangle Biomedical Sciences, Durham, NC Cat# TFM-5) and 20 µm coronal (transverse) spinal cord sections were prepared using a cryostat and stored at -20C. After thawing, non-specific binding block was applied (5% BSA, 10% normal donkey serum (NDS), 0.2% Triton X-100 in PBS, 1 hr at room temperature) followed by a previously described immunostaining protocol (Saraswat Ohri et al., 2011, 2018, Bankston et al., 2019). Primary antibodies used were as follows: anti-APC (species: mouse CC1 clone, 1:200, Abcam, Cat# ab16794, RRID: AB_443473), anti-HA (species: mouse, 1:1000, HA.11 Clone 1612, Biolegend, Cat# 901516, RRID: AB_2820200), anti-STEAP3 (species: rabbit, dilution: 1:200, Thermo Fisher Scientific Cat# PA5-102321, RRID: AB_2851729), anti-phospho-NFH (species: mouse, dilution: 1:500, Biolegend Cat# 801601, RRID: AB_2564641), anti-MBP (species: chicken, dilution: 1:500, Thermo Fisher Scientific Cat# PA1-10008, RRID: AB_1077024), anti-PCYOX1L (species: rabbit, dilution: 1:200, Atlas Antibodies, Bromma, Sweden, Cat# HPA037463, RRID: AB_10673632), and anti-CNP (species: mouse, dilution: 1:200, Biolegend Cat# 836404, RRID AB_2566639). Species- and isotype-specific Alexa488-, Alexa594-, or Alexa647- F(ab')2 secondary antibodies (donkey, 1:200, Life Technologies) were used for fluorescent visualization of protein labeling. Hoechst-33258 as used to counter-stain nuclei. Negative controls were performed using species-specific non-immune Ig subtypes in place of primary antibodies. For quantification of Ribotag expression, all images were captured with a Nikon TE 300 inverted microscope (Nikon) equipped with a Spot CCD camera using identical exposure settings. Images were photographed with a 10X, 20X or 40X objective as indicated in the figure legends and stitched with Elements™ software during acquisition. Confocal imaging of STEAP3 and PCYOX1L immunostaining was

performed using a Nikon C2+ confocal microscope with intelligent acquisition, deconvolution, 3D measurement package, artificial intelligence image processing, LUN4 solid state laser launch (405, 488, 561, 640 nm), DUVb high-sensitivity GaAsP detectors, perfect focus system and an ORCA-FUSION Gen-III sCMOS monochrome camera.

Immunoblotting. Protein lysates were prepared in 10mM Tris pH 7.4, 0.2% Triton X-100 (+protease inhibitor cocktail) using a microtube homogenizer on ice. Following protein concentration measurement (BCA assay, Pierce), equal amounts of total protein (15-20 μg/sample) were separated using SDS-PAGE and transferred to PVDF membrane (Millipore, Burlington MA). Blot processing followed standard methodology with primary antibody incubations performed at 4°C overnight.

Supplementary Methods Table SM1. Design of the qPCR custom microfluidic card (Custom TaqMan Gene Expression Array Cards (Thermo Fisher, catalog # 43442249)).

Gene	Gene description	Assay ID: Custom TaqMan Gene Expression Array Card Cat# 43442249	cell type marker
Mbp	myelin basic protein	Mm01266402_m1	OL
Plp1	proteolipid protein (myelin) 1	Mm01297210_m1	OL
Mog	myelin oligodendrocyte glycoprotein	Mm01279062_m1	OL
Cldn11	claudin 11	CMm00500915_m1	OL
Mobp	myelin-associated oligodendrocytic basic protein	Mm02745649_m1	OL
Opalin	oligodendrocytic myelin paranodal and inner loop protein	Mm00463365_m1	OL
Mag	myelin-associated glycoprotein	Mm00487538_m1	OL
Fa2h	fatty acid 2-hydroxylase	Mm00626259_m1	OL
Gjb1	gap junction protein, beta 1	Mm01950058_s1	OL
Ermn	ermin, ERM-like protein	Mm01331072_m1	OL
Gjc2	gap junction protein, gamma 2	Mm00519131_s1	OL
Klk6	kallikrein related-peptidase 6	Mm00478322_m1	OL
Sox10	SRY (sex determining region Y)-box 10	Mm00569909_m1	OL
Aldh1l1	aldehyde dehydrogenase 1 family, member L1	Mm03048957_m1	astrocyte
Hgf	hepatocyte growth factor	Mm01135184_m1	astrocyte
Reln	Reelin	Mm00465200_m1	neuron
Snap25	synaptosomal-associated protein 25	Mm01276449_m1	neuron
Lhx5	LIM homeobox protein 5	Mm00521778_m1	neuron
Osm	oncostatin M	Mm01193966_m1	microglia
Cd68	CD68 antigen	Mm03047343_m1	microglia
Tmem119	transmembrane protein 119	Mm00525305_m1	microglia
Ppia	Eukaryotic 18S rRNA	Mm02342430_g1	all cell control
Ywhaz	peptidylprolyl isomerase A	Mm03950126_s1	all cell control
18S	tyrosine 3-monooxygenase/tryptophan 5-monooxygenase activation protein, zeta polypeptide	Hs99999901_s1	all cell control

Supplementary Figure Legends

Supplementary Fig. S1: Successful Isolation of the OL translatome after SCI. OL-enriched transcripts (Fig. 2, Supplementary Table S1) with enrichment Log2FC(Total)>2, q<0.05 were analyzed for GO term overrepresentation. The top 10 most overrepresented gene ontology terms (GOs) are shown for dpi 2 ***(a)***, 10 ***(b)*** and 42 ***(c)***. GOs related to OL function (red boxes) show a high level of overrepresentation. Full results of GO analysis are in Supplementary Table S2.

Supplementary Fig. S2: Cholesterol biosynthesis pathway. A simplified superpathway of cholesterol biosynthesis was created using the KEGG pathways mmu:00100: steroid biosynthesis and mmu:00900: terpenoid backbone biosynthesis. Due to the complexity of the pathways, only genes listed in Fig. 5c are included. Genes in green are downregulated in OLs on dpi 10 and 42. Only a single component of the superpathway, Hmgcs2 is upregulated on dpi 2, 10, and 42 (red). Unlabeled arrows represent cholesterol biosynthesis steps whose enzymes/genes show unaffected OL expression after SCI. Note SCI-associated OL downregulation of the critical regulators of cholesterol biosynthesis: *Hmgcr* and *Sqle*.

Supplementary Figure S3. STEAP3 expression is upregulated in the ventral and lateral white matter after acute SCI. ***(a-b)*** Low magnification images of spinal cord sections with or without SCI that were immunostained for STEAP3 (tissue preparation and co-immunostainings as described for Fig. 9). Specificity controls with species-

matched non-immune IgGs that were used instead of primary antibodies are shown in *(a)*.

Supplementary Figure S4. STEAP3 expression on dpi 1. Tissue preparation and co-immunostainings as described for Fig. 9. Inset boxes indicate the ventrolateral white matter regions analyzed. ***(a, c)*** Specificity controls with species-matched non-immune IgGs that were used instead of primary antibodies. ***(b)*** Arrows identify $CC1^+$ cells with positive STEAP3 signal. Arrowheads mark $STEAP3^+$ structures that are not associated with $CC1^+$ cell somas and/or $DAPI^+$ nuclei. Such structures appear as the main source of increased STEAP3 staining in the white matter of the injured spinal cord. Z-axis profiles show representative CC1+ cell soma signals for STEAP3 (cross). ***(d)*** Most STEAP3 signal is found adjacent to distended, damaged axons that are identified by positive p-NFH immunostaining (arrowheads).

Supplementary Figure S5. STEAP3 is upregulated acutely after SCI *in vivo* and in the OPC lineage in response to mitochondrial uncoupling *in vitro.* Original full-length images of cropped blots presented in figure 8. ***(a)*** STEAP3 is significantly upregulated during acute SCI (dpi 2) but is not at later timepoints (dpi 10 and 42). Predicted kDa of STEAP3 is ~55 kDa. Actual size seen with the anti-STEAP3 antibody, which targets the N-terminal region of STEAP3, shows a slightly higher molecular weight in the mouse spinal cord tissue ***(a)***. A similar size band is shown by the manufacturer in the mouse liver (Cat #: PA5-102321). ***(b,c)*** STEAP3 is upregulated in response to the mitochondrial uncoupler FCCP in cultured rat OPCs and OPC-derived

OLs. The anti-STEAP3 antibody detects a band with a slightly lower molecular weight than the expected 55 kDa or the STEAP3 band in the mouse spinal cord tissue (a). This likely due to differences in protein processing and/or additional post-translational modifications. Of note, slightly lower than expected molecular weight of STEAP3 was observed with another anti-STEAP3 antibody that was raised against the N-terminal region in human HeLa cells (Thermo Fisher Scientific, Cat #: PA5-20406, manufacturer's data). In (b, c), lanes containing samples that were unrelated to the current study are marked (Cell X, Drug X); those lanes were removed from blot panels that are shown in Fig. 8.

Supplementary Figure S6. OL expression of PCYOX1L before and after SCI. ***(a)*** WT naïve and dpi 2 SCI tissue co-immunostained with mouse anti-CC1, a marker for mature OLs, and rabbit anti-PCYOX1L. Confocal images confirm colocalization of PYCOX1L+ and CC1+ expressing OLs in naïve and dpi 2 animals. PCYOX1L expression increases in dpi 2 tissue in the ventral and lateral white matter. ***(b)*** WT naïve and dpi 2 SCI tissue co-immunostained with mouse anti-CNP, a marker for premyelinating and myelinating OLs, and rabbit anti-PCYOX1L further confirms PCYOX1L expression after SCI. Arrows indicate $CC1^+$ or CNP^+ OLs that show positive PCYOX1L signal in their cell somas and soma-proximal processes. Such cells are present in both control- and SCI tissue. Somatic, vesicular/granule-like appearance of PCYOX1L signal is consistent with its expected localization to lysosomes. Similar cell soma/process localization of PCYOX1L+ staining was also observed on dpi 1 (data not shown). Staining specificity controls are shown in Supplementary Figs S3 and S4.

Supplementary Figure S7. Unique disease-associated OL transcriptome profiles are overrepresented in OL translatome-upregulated mRNAs after SCI in a time-dependent manner. Overrepresentation factor of the OL translatome overlaps with single cell RNASeq-derived OL transcriptomic profiles that are common for various types of white matter damage (PMID: 36001972, http://research-pub.gene.com/OligoLandscape/, see discussion for details). The DA1 pro-inflammatory cluster shows the greatest overlap with the OL SCI translatome reaching a maximum on dpi 10.

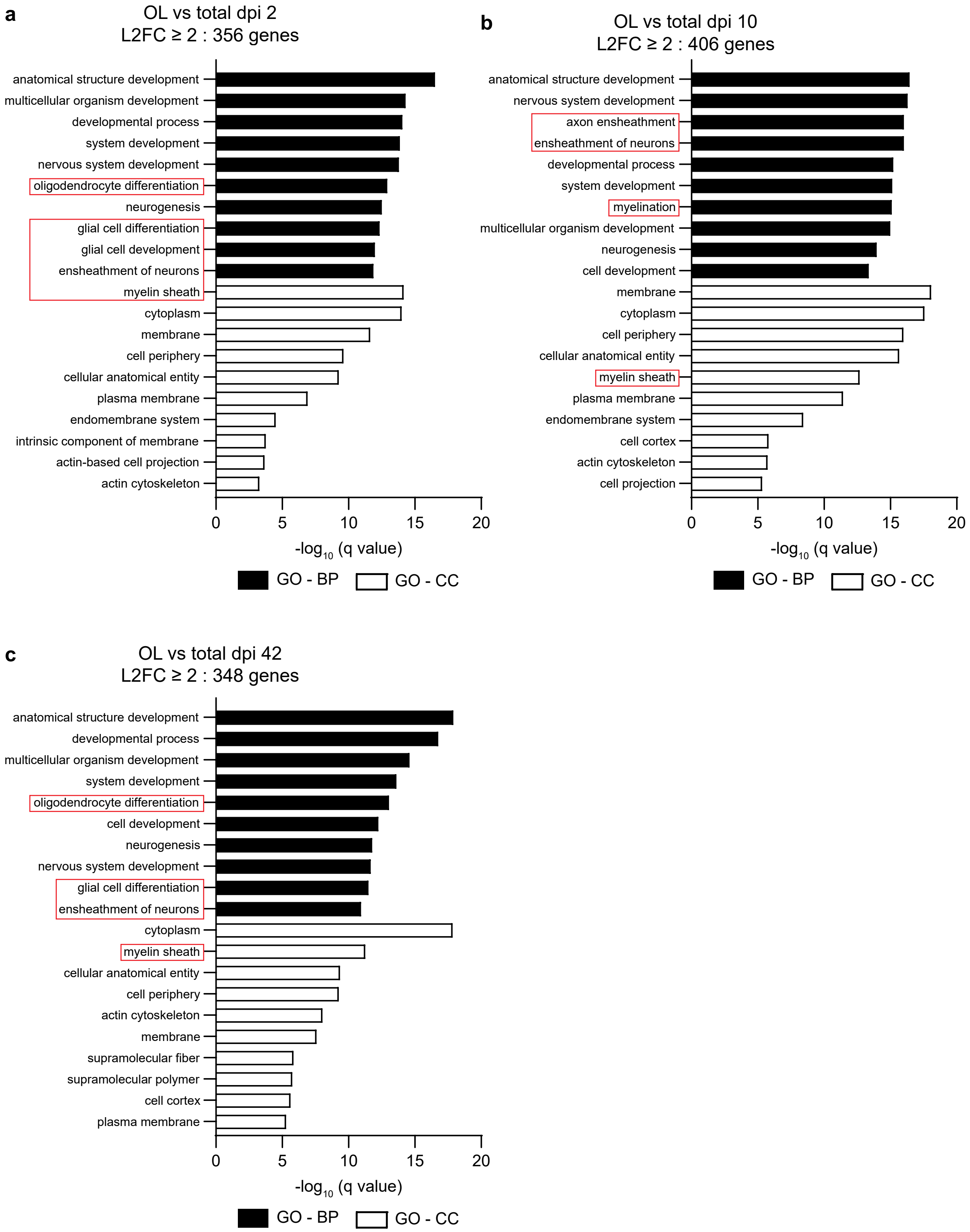

Forston et al. Supplementary Fig. S1

cholesterol biosynthesis pathway

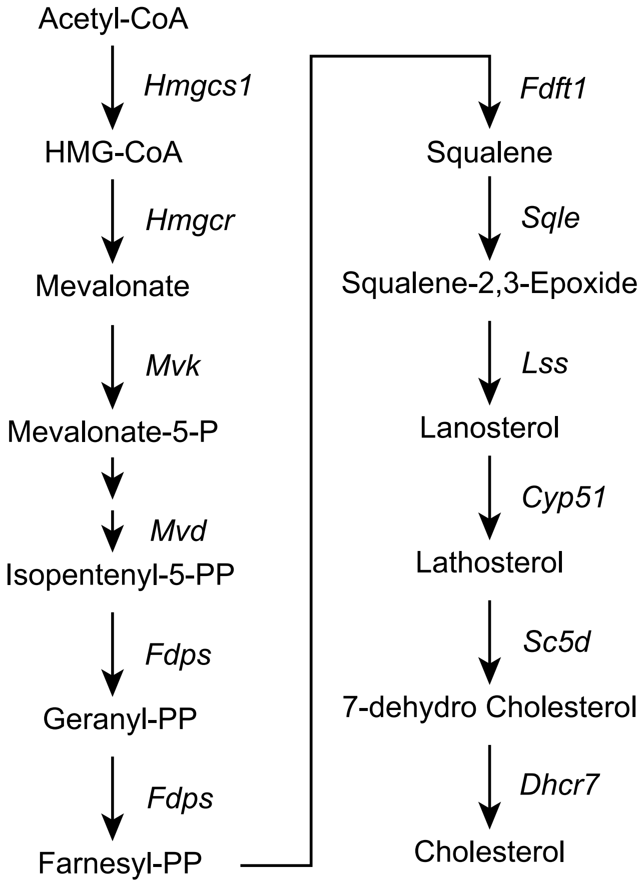

Forston et al. Supplementary Fig. S2

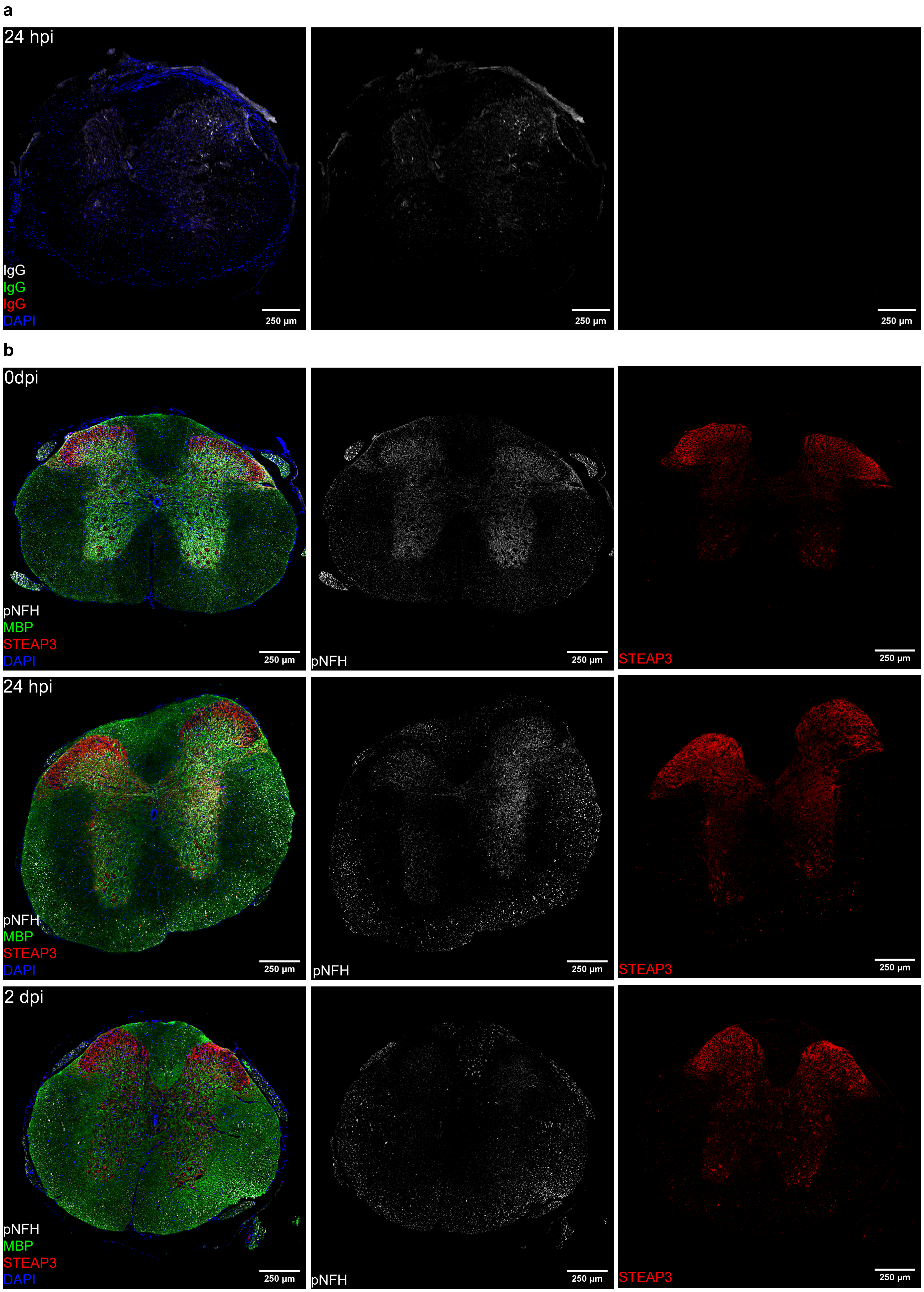

Forston et al. Supplementary Fig. S3

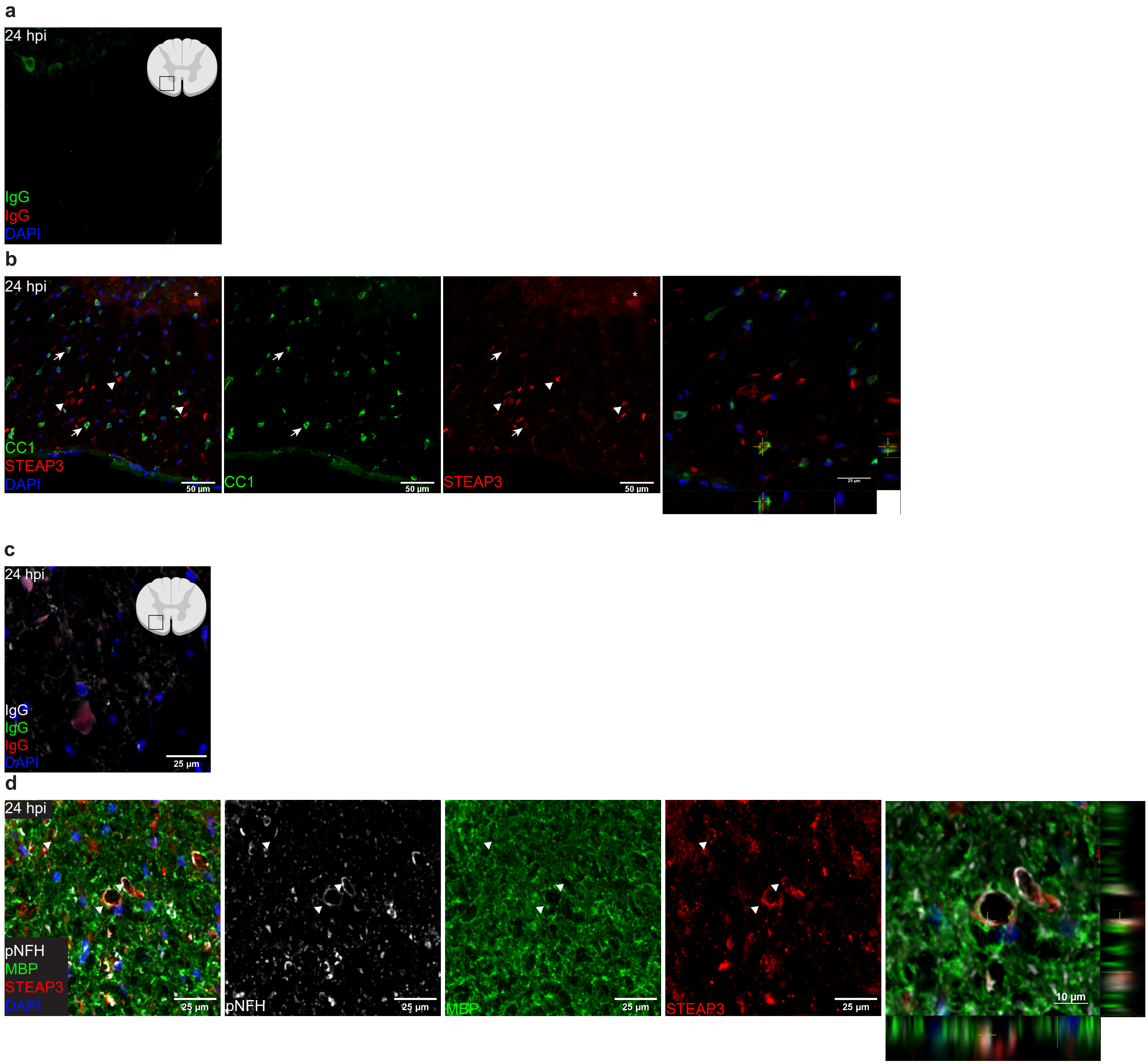

Forston et al. Supplementary Fig. S4

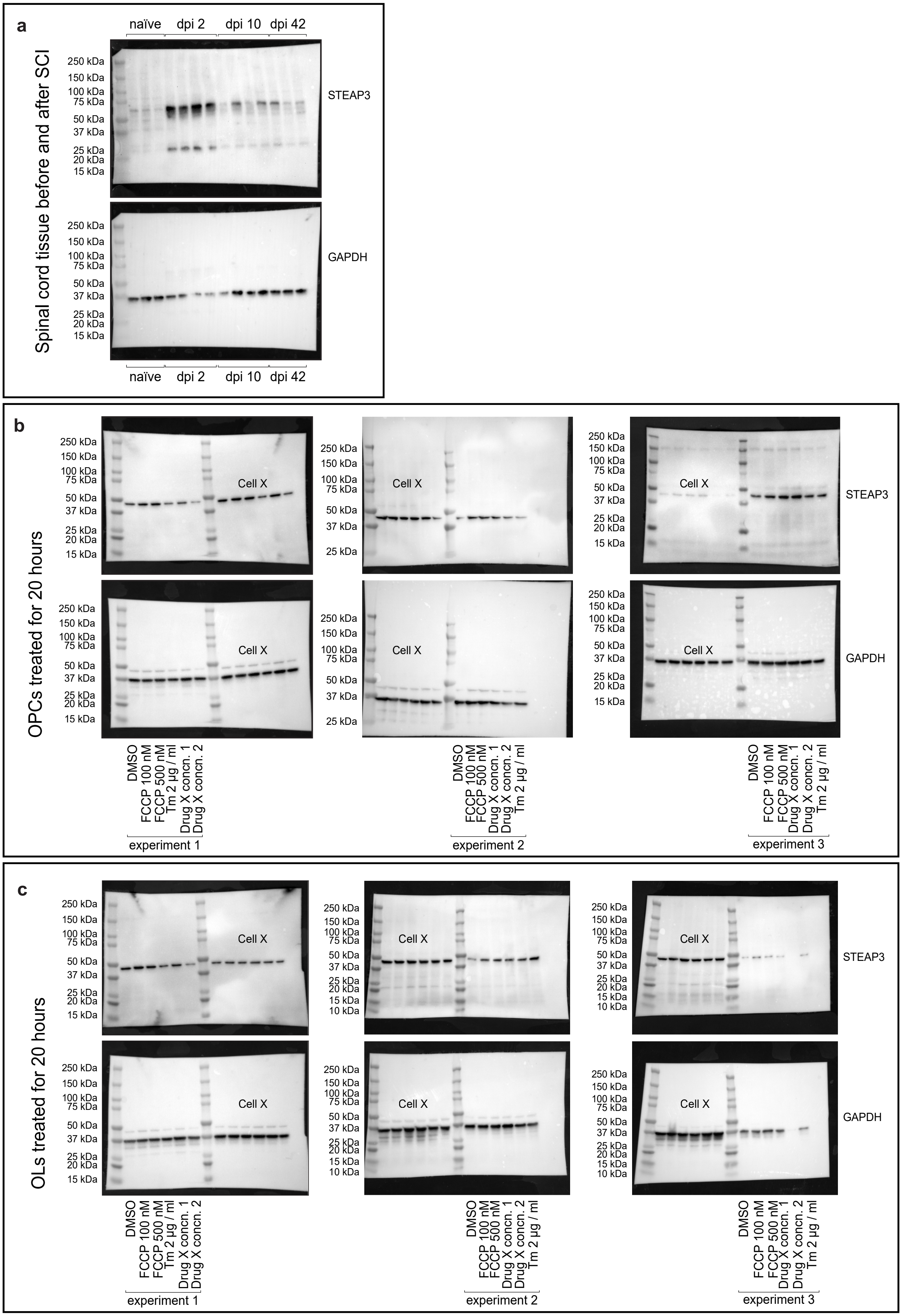

Forston et al. Supplementary Fig. 5

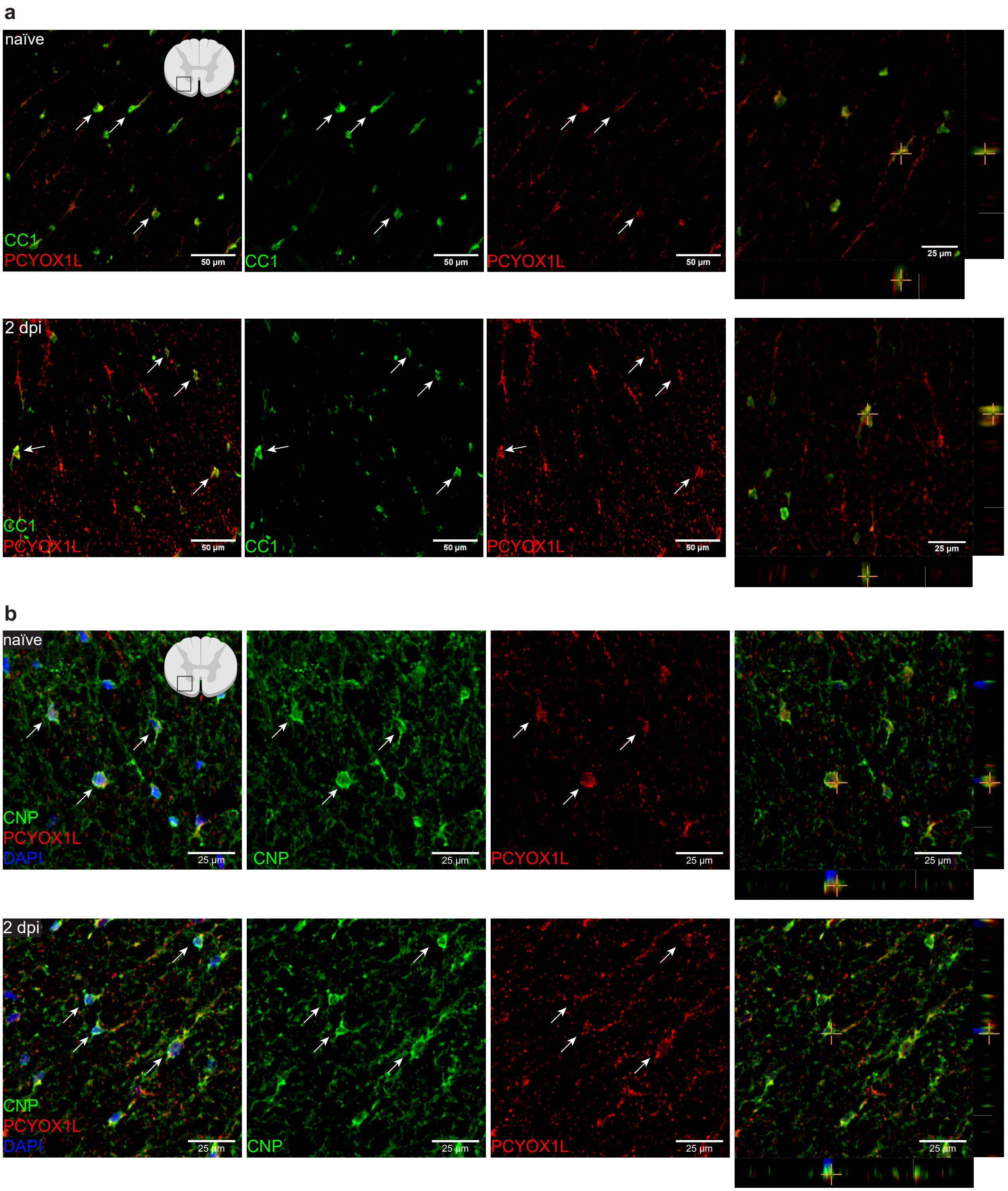
a
naïve
CC1
PCYOX1L
50 µm
25 µm
2 dpi
b
CNP
DAPI

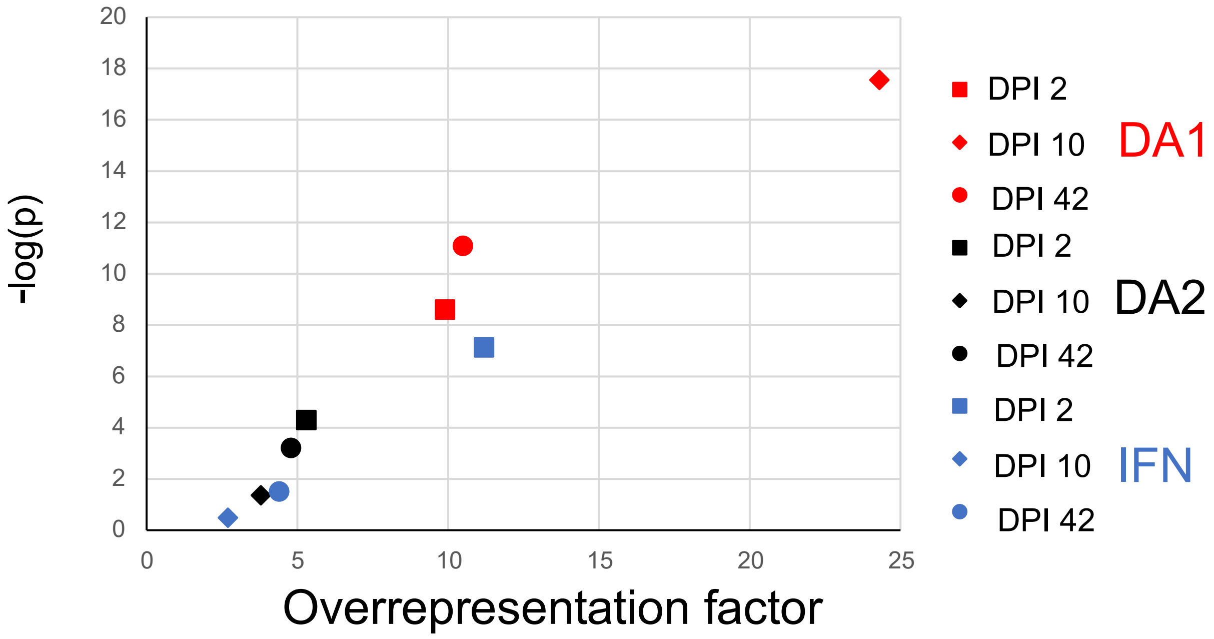
20
18
16
14
12
10
8
6
4
2
0
0
5
10
15
20
25
-log(p)
Overrepresentation factor
DPI 2
DPI 10
DA1
DPI 42
DPI 2
DPI 10
DA2
DPI 42
DPI 2
DPI 10
IFN
DPI 42

List of Supplementary Tables:

Supplementary Table S1. OL enriched mRNAs in mouse spinal cord tissue (Log_2FC/Total/>0.5, q<0.05)

Supplementary Table S2. GO analysis of OL translatome-enriched mRNAs in naive, dpi 2, 10 and 42 mice

Supplementary Table S3. Supplementary Table S3. Differentially expressed OL translatome mRNAs in the contused spinal cord (|Log2FC/naïve/|>1, q<0.05, filtered for differential OL enrichment or constantly high OL enrichment)

Supplementary Table S4. GO analysis of OL translatome genes that are highly upregulated or downregulated after SCI (|Log_2FC/naive/|>1, q<0.05)

Supplementary Table S5. OL-upregulated mRNAs whose expression pattern correlates with acute OL loss and tissue damage after SCI: gene lists and GO analysis.

Supplementary Table S6. Operon enrichment among OL-upregulated genes on dpi 2, 10, or 42
